# Public health impacts of city policies to reduce climate change: findings from the URGENCHE EU-China project

**DOI:** 10.1186/s12940-016-0097-0

**Published:** 2016-03-08

**Authors:** Clive E. Sabel, Rosemary Hiscock, Arja Asikainen, Jun Bi, Mike Depledge, Sef van den Elshout, Rainer Friedrich, Ganlin Huang, Fintan Hurley, Matti Jantunen, Spyros P. Karakitsios, Menno Keuken, Simon Kingham, Periklis Kontoroupis, Nino Kuenzli, Miaomiao Liu, Marco Martuzzi, Katie Morton, Pierpaolo Mudu, Marjo Niittynen, Laura Perez, Denis Sarigiannis, Will Stahl-Timmins, Myriam Tobollik, Jouni Tuomisto, Saskia Willers

**Affiliations:** School of Geographical Sciences, University of Bristol, Bristol, BS8 1SS UK; National Institute for Health and Welfare, FI-70701 Kuopio, Finland; School of the Environment, Nanjing University, Nanjing, 210023 China; European Centre for Environment and Human health, University of Exeter Medical School, Exeter, EX1 2LU UK; Air Quality Department, DCMR Environmental Protection Agency Rijnmond, Schiedam, The Netherlands; Institute of Energy Economics and the Rational Use of Energy (IER), University of Stuttgart, 70565 Stuttgart, Germany; IOM (Institute of Occupational Medicine), Riccarton, Edinburgh, Scotland UK; National Institute for Health and Welfare, 70210 Kuopio, Finland; Centre for Research and Technology Hellas, Chemical Process and Energy Resources Institute, 57001 Thermi, Greece; Netherlands Organization for Applied Research (TNO), 3584 CB Utrecht, The Netherlands; Department of Geography, University of Canterbury, Christchurch, New Zealand; Swiss Tropical and Public Health Institute (Swiss TPH), University of Basel, Basel, Switzerland; European Centre for Environment and Health, WHO Regional Office for Europe, 53113 Bonn, Germany; Environmental Engineering Laboratory, Department of Chemical Engineering, Aristotle University of Thessaloniki, Thessaloniki, 54124 Greece; The BMJ, BMA House, London, WC1H 9JP UK; School of Public Health, University of Bielefeld, Bielefeld, Germany

**Keywords:** Greenhouse gas emission reduction policies, Health, Wellbeing, Urban, Europe, China, Air pollution, Transport, Buildings, Energy

## Abstract

**Background:**

Climate change is a global threat to health and wellbeing. Here we provide findings of an international research project investigating the health and wellbeing impacts of policies to reduce greenhouse gas emissions in urban environments.

**Methods:**

Five European and two Chinese city authorities and partner academic organisations formed the project consortium. The methodology involved modelling the impact of adopted urban climate-change mitigation transport, buildings and energy policy scenarios, usually for the year 2020 and comparing them with business as usual (BAU) scenarios (where policies had not been adopted). Carbon dioxide emissions, health impacting exposures (air pollution, noise and physical activity), health (cardiovascular, respiratory, cancer and leukaemia) and wellbeing (including noise related wellbeing, overall wellbeing, economic wellbeing and inequalities) were modelled. The scenarios were developed from corresponding known levels in 2010 and pre-existing exposure response functions. Additionally there were literature reviews, three longitudinal observational studies and two cross sectional surveys.

**Results:**

There are four key findings. Firstly introduction of electric cars may confer some small health benefits but it would be unwise for a city to invest in electric vehicles unless their power generation fuel mix generates fewer emissions than petrol and diesel. Second, adopting policies to reduce private car use may have benefits for carbon dioxide reduction and positive health impacts through reduced noise and increased physical activity. Third, the benefits of carbon dioxide reduction from increasing housing efficiency are likely to be minor and co-benefits for health and wellbeing are dependent on good air exchange. Fourthly, although heating dwellings by in-home biomass burning may reduce carbon dioxide emissions, consequences for health and wellbeing were negative with the technology in use in the cities studied.

**Conclusions:**

The climate-change reduction policies reduced CO_2_ emissions (the most common greenhouse gas) from cities but impact on global emissions of CO_2_ would be more limited due to some displacement of emissions. The health and wellbeing impacts varied and were often limited reflecting existing relatively high quality of life and environmental standards in most of the participating cities; the greatest potential for future health benefit occurs in less developed or developing countries.

**Electronic supplementary material:**

The online version of this article (doi:10.1186/s12940-016-0097-0) contains supplementary material, which is available to authorized users.

## Background

Globally, our climate is changing due to anthropogenic activity; this is already having, and will increasingly have, serious consequences for human and natural systems; urban areas are at a particularly high risk [1]. However urban areas are also responsible for producing more than 70 % of the greenhouse gas (GHG) emissions [2] which are changing the climate. For these reasons this paper describes an assessment of policies that governance bodies responsible for urban areas, such as city councils, can instigate in order to reduce GHG emissions.

There has been increasing interest in the implications of such urban GHG reduction policies for health, notably respiratory and cardio-vascular outcomes [3]. Climate change also has implications for wellbeing [4] but less attention has been paid to the impact of mitigation measures on wellbeing. The definition of wellbeing remains contested but the World Health Organization (WHO) Regional Office for Europe has proposed: “an individual’s experience of their life as well as a comparison of life circumstances with social norms and values” [5]. Wellbeing involves positive mental health, adequate resources for living a fulfilled life and a society with high levels of wellbeing is likely to have low levels of inequalities [6, 7]. GHG reduction policies which provide positive co-benefits for health and wellbeing are more likely to be acceptable to governing bodies and their populations.

China has the highest carbon dioxide (CO_2_) emissions globally [8] (CO_2_ makes up the largest proportion of GHG [9]) and Europe is leading the way in mitigation [10]. This paper describes the findings of an EU FP7 funded project, Urban Reduction in Greenhouse Gas Emissions in China and Europe (URGENCHE). The primary objective of this work was to quantify the co-benefits of policies to mitigate climate change in urban areas whilst simultaneously improving citizen’s health and wellbeing. Whilst there is an extensive literature on health impact assessment (HIA) for traditional mortality and morbidity health outcomes, rarely has wellbeing and quality of life been addressed. In this paper we illustrate the progress the URGENCHE project has made in these areas, both theoretically and substantively.

## Methods

**Fig. 1:**
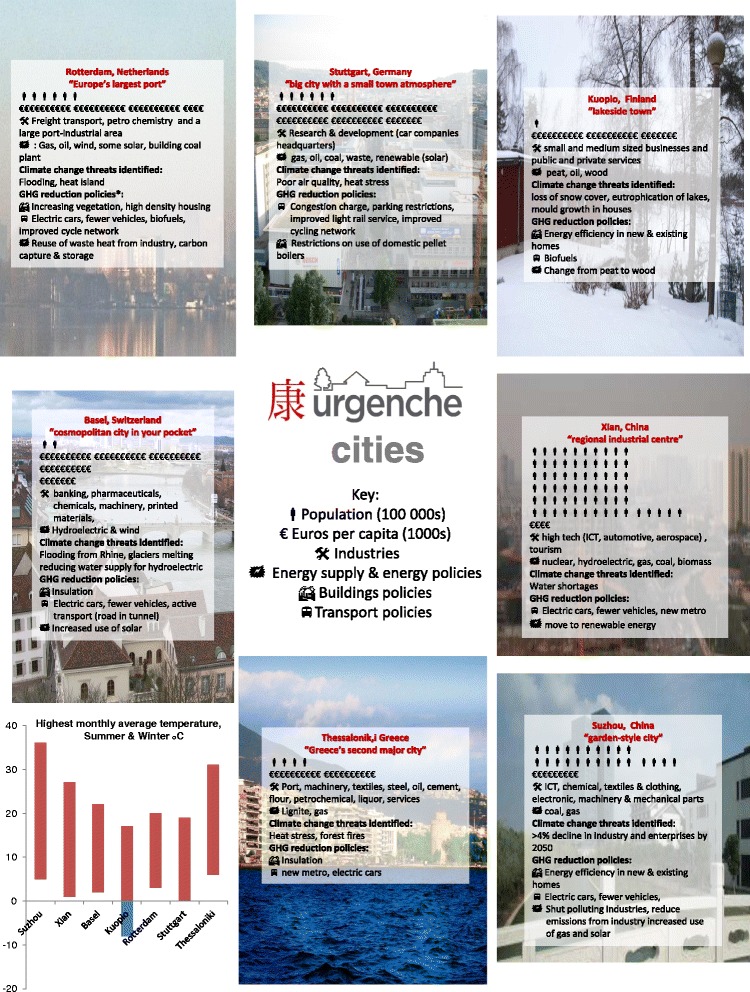
City context and policies examined

**Fig. 2:**
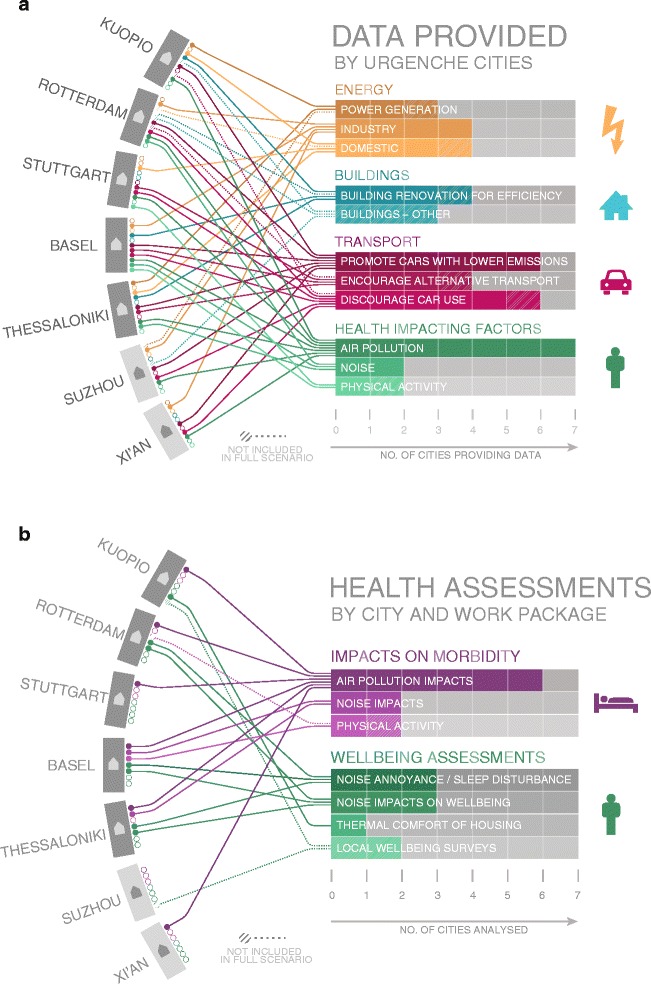
City data provision. (**a**) Energy, Buildings, Transport and Health impacts (**b**) Health assessments

Seven cities, each with a partner academic team, were recruited to URGENCHE, five from Europe (Kuopio, Finland; Rotterdam, Netherlands; Stuttgart, Germany; Basel, Switzerland and Thessaloniki, Greece) and two from China (Xi’an and Suzhou); the cities varied greatly in terms of Gross Domestic Product (GDP), climate, population, climate change threats and mitigation policies (Fig. 1). The academics were internationally recognised scientists in the areas of geography, health risk assessment, urban energy demand and supply scenarios, urban planning, environmental science and epidemiology. Several approaches were employed to produce an original combination of methods applied at an urban scale. The main methodology was the development of scenarios and assessing comparisons between them. However a flexible approach was taken and methodologies were adapted to suit the needs and interests of individual cities and participants. The data collated and health and wellbeing impacts assessed via URGENCHE differed for each city (Fig. 2a, b). Next more detail about the URGENCHE methodologies is described followed by the methods used to collate results for this paper.

### Scenario development

**Fig. 3:**
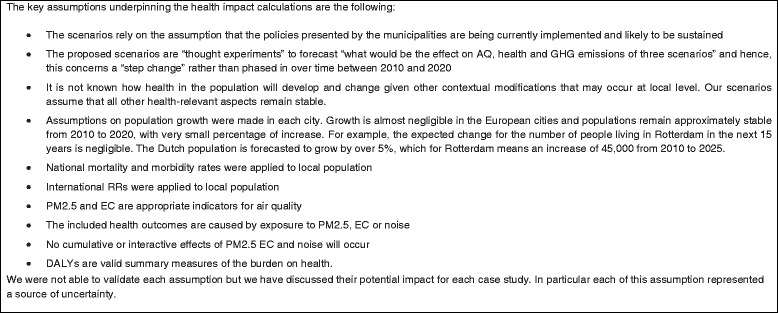
Health impact assumptions

The scenarios examined three broad domains: urban transport; building fabric and energy supply. Potential effects of policies on human health were estimated by following the impact assessment approach. A GIS-based methodology took into account the advances made in health integrated assessment in a large range of studies in Europe over recent years. Policies focussed on levels of different fuels used in power generation, energy efficient buildings in the cities and transport modes because these impact on GHG emissions and health and wellbeing. Levels of CO_2_ emissions, health impacting exposures (cities chose one or more of air pollution, noise and physical activity) and health and wellbeing in 2010 were established for each city. This information, atmospheric models and exposure response functions (ERF), derived from epidemiological studies (especially the WHO HRAPIE set of functions [11] and noise ERFs developed by Miedema and collegues [12–14]), were used to model relationships between health impacting factors and health and wellbeing in 2020, together with information about expected changes such as industry development, population growth and consequent building activity, traffic growth, and adoption of the increasingly stringent traffic emission standards. The key assumptions underpinning the health impact assessments are presented in (Fig. 3). Uncertainty was considered and discussed in each step of the project and in each health impact assessment.

The expected levels of CO_2_ emissions, health impacting exposures and health and wellbeing in 2020 were known as a ‘business as usual’ (BAU) scenario. In some cities where GHG emission reduction measures had already been commissioned these were also included. Note that the dates 2010 and 2020 were flexible if this was helpful to the cities but cities tended to use these, or dates within 5 years, for most of their work.

City council personnel and local academics associated with URGENCHE were asked to provide some additional hypothetical GHG reduction policies to model in order to understand whether outcomes in 2020 would differ if these policies were enacted compared with the BAU scenarios. In general city-specific policies were applied but there was one instance where the impacts of two identical policies (10 % reduction in private car use and 50 % growth in electric cars) were modelled across four cities [15].

Innovative methodologies were needed in order to produce these scenarios and these are now able to be shared as modelling tools. Tools to calculate the contribution of various sources of power to cities’ electricity and heating [16] and impacts of building policies [17] are available via open-source wikis.

Our methodology was highly data dependant. Lack of suitably spatially resolved data was a particular problem for the Chinese cities and for wellbeing. If data for modelling was not available for a city, open source data [15] or data for a whole region [18] or a similar city [19] were used as a substitute. For wellbeing a methodology was established to link the European Quality of Life Survey (EQLS) data [20] on wellbeing and noise annoyance to noise level changes modelled in city scenarios [18].

### Comparative risk assessment (CRA) approach to health measurement

We used a CRA approach based on the Population Attributable Fraction that is the fraction by which the occurrence of a disease of interest would be reduced under an alternative, usually more favourable, exposure distribution. Disability-adjusted life-years (DALYs) were calculated using country-levels profiles, as city-specific profiles were not available.

DALYs were calculated as the sum of Years of Life Lost (YLL) gained (or lost) due to deaths prevented (or brought forward) and additional years of life lost due to disability (YLD) from outcomes related to health and well-being aspects (Restricted Activity Days (RADs) for air pollution exposure and annoyance and sleep disturbance for noise). In the cities, YLDs were estimated using a disability weight of 0.02 per year for both outcomes as recommend in the WHO noise health impact guidelines. No discounting in years of age was used. Effects of exposures were differentiated – for example effects of noise exposures on mortality, annoyance and sleep disturbance were calculated separately. Our approach was to minimize double counting of impacts, but it has to be recognized that the calculated benefits may correspond to an underestimation of the total benefits [21, 22].

### Literature reviews

Four literature reviews were conducted. Firstly, a literature review was conducted in order to establish health and wellbeing impacts of climate change itself [23]. This put the URGENCHE results into perspective. The second literature review was a scoping exercise to understand the concept of wellbeing and how it could be impacted by GHG reduction measures [6]. The third and fourth literature reviews were intended to extract ERFs between health and wellbeing impacting factors (air pollution, noise and/or physical activity) and health and wellbeing; these results informed the scenario development and analysis. Box A2 in Additional file 1 provides the ERFs used for noise.

### Wellbeing surveys

The paucity of literature on wellbeing and the lack of city data on wellbeing engendered the development of cross sectional surveys which were undertaken in Kuopio (*n* = 782) and Suzhou (*n* = 775). Ethical permission was granted. Details are provided in Additional file 1 (box A3).

### Longitudinal studies

The lack of data available in some urban environments in China led to the development of a longitudinal study on current conditions in Suzhou [24]. In Rotterdam, analysis was undertaken to understand the relationship between heat stress, air pollution and mortality in a European country where temperatures are less extreme than some other parts of the world currently. However, higher temperatures are likely due to climate change [25]. In Thessaloniki a tax on fossil fuels was introduced (due to the Greek economic crisis) during the URGENCHE project period and the opportunity was taken to gather data on its impacts [26].

### Methodology for this paper

The aim of this paper was to bring together the results in order to make some overarching conclusions about the impacts of the policies studied. The first type of impact collated was the impact of CO_2_ emissions in order to find out whether the policies were likely to mitigate the effects of climate change. The second impact collected was the impact of health impacting factors (air pollution, noise and physical activity). The third impact studied was the impact on morbidity and mortality and the fourth impact studied was the impact on wellbeing. The impact on wellbeing had three elements: firstly impact on wellbeing specific to the environmental change e.g. noise annoyance, secondly the impact on overall wellbeing and thirdly the impact on economic wellbeing. Economics are of increasing import particularly since the recent recession [26] and indeed sometimes wellbeing has been viewed purely in an economic sense [27]. Finally results indicating impacts of the policies on health inequalities were collected. It is important that the impacts of GHG reduction policies are seen in context. Thus at the start of each section on health and wellbeing, results from our literature review on the impacts of climate change on wellbeing are presented [23]. A table summarising findings about all policies studied on emission reduction and health and wellbeing is also included.

Given the city-centric approach of URGENCHE, the results were presented in various formats. In order to provide an overview the conventions used are provided in Box A1 (see Additional file 1). Each comparison in the tables (see Additional file 1) is comparing the impact in around the year 2020 of adopting a policy with not adopting a policy (usually BAU), unless otherwise stated. Exposures and impacts are disaggregated where possible.

## Results

Detailed results tables (Additional file 1: Tables A1 to A4) are presented in the additional file and the summary table is included here [Table 1].

### Carbon dioxide emissions

If the future develops in a ‘business as usual’ (BAU) manner, global emission reduction targets, for instance to keep temperature rises below 2 °C [28] will not be met. In the traffic sector for example, BAU models suggest that European emissions would be similar in 2010 and 2020 but will increase by about 40 % in Chinese cities [15]. All policies modelled reduced CO_2_ emissions (Additional file 1: Tables A1a and A1b) but there was only a minor reduction in emissions from domestic wood burning in Kuopio and promotion of public and active transport in Stuttgart. Furthermore although the policies would reduce emissions it was not necessarily the case that global emissions would consequently reduce. In some instances other factors compensated for the reduction: for two of three scenarios envisaging housing efficiency improvements, the reduction in heat demand was outweighed by the projected number of new buildings [29]; similarly the electricity needed to produce geothermal energy reduces its net output [30]. In other cases despite a reduction in emissions from a city, global emissions might not decrease because emissions had simply been transferred elsewhere. Suzhou Synthetic Chemical Co. Limited, for example, moved from the urban area of Suzhou to Zhangjiagang in 2003 [31]. Biomass can be associated with GHG emissions through fertiliser application, deforestation and transport [32]. Biofuels have similar issues with agricultural and transport emissions [30, 33]. Additionally global emissions will not be reduced if discouraging heavy industry displaces it geographically [24] or if electricity to power electric cars is created through fossil fuels.

The policy which appeared to be responsible for a significant reduction in CO_2_ emissions in Stuttgart that was not offset or context dependent was the introduction of a congestion charge. Models suggested this would have a greater impact on citizens’ transport mode choice than provision of enhanced infrastructure for public or active transport or parking management. Models of two other policies suggested they could lead to substantial reductions in CO_2_ emissions but both of these were more context dependent. First was changing a power plant’s fuel from peat to wood. Few power plants globally however rely on peat and for wood to be a good substitute there needs to be a plentiful local supply in sustainably managed forests. Peat is an important energy source only in Finland and Ireland (where 5–7 % primary energy consumption relies on peat) and to a lesser extent in Sweden and the Baltic states [34, 35]. The second policy was introducing electric cars – however this was only certain to reduce overall emissions substantially in Basel where electricity power production is already 100 % renewable.

Additionally some policies modelled were deliberately unrealistic in order to understand the theoretical maximum effect on CO_2_ or other air pollutants’ emissions including 100 % pre 1980 buildings renovated [29], 50 % cars fuelled by electricity [21] and all post 2010 oil and wood powered residences changed to solar/wind (except 50 % of electric powered residences) [30]. Furthermore there may be a limit to these mitigation policies: for example Suzhou is approaching European levels of clean technology [24] and 100 % renewable energy is used to generate electricity for Basel and so capacity for further reduction is limited.

We had expected to model scenarios with larger proportions of city energy mix from solar/wind/hydro. By working with city partners we found that there was actually much more interest in biomass (chiefly wood) burning. When researching our methodology we found conflicting advice in the literature as to the consequences of biomass combustion for CO_2_ emissions: in some cases biomass combustion was viewed as greenhouse neutral because carbon is absorbed by the plant and then released by combustion to be absorbed by future plants in a cycle but its direct emission factor is 420 kg/MWh [29]. Thus our results may overestimate the effectiveness of biomass in emission reduction. Biomass is not recommended unless the GHG emissions are 60 % lower than the energy carriers it is replacing [32].

#### Interim conclusions

Reduction in CO_2_ emissions from substitution of fossil fuels by electricity will be offset by emissions from fossil fuels used to generate electricity. Thus we recommend addressing energy mix as a first step. The optimum city energy mix is strongly context dependent and not all cities will either have control of their mix or, even if they have control, the resources to switch to less carbon-intensive sources. Other cities, for example our case study city of Basel, have the potential to create a surplus of renewably generated electricity. Thus we support the creation of a European Energy Union [36] which is intended to enable transfer of surplus renewably generated energy.

City planners should take into account spatial displacement of CO_2_ emissions in their calculations. Reducing city emissions may lead to higher emissions elsewhere. For climate change mitigation to occur it is necessary to reduce emissions globally. Thus calculations should not be limited to within city boundaries. Additionally city planners need to take into account that city growth may offset modest reductions in CO_2_ emissions from policies. Substantial changes need to be made to energy mix, buildings and transport mode before CO_2_ emissions will be reduced. In terms of transport, we recommend introducing a congestion charge as a successful and realistic way of reducing CO_2_ emissions.

### Health impacting exposures

#### Air pollution

Local policies (for example renovating building stock, changing the fuel mix of local power plants, discouraging car use and promoting other forms of transport and in general promoting electric cars) were found to have limited effects on reducing local ambient air pollution levels (Additional file 1: Table A2a). Even though some policies, such as changing the Kuopio power plant fuel source from peat to wood and introducing a congestion charge in the Stuttgart area, were estimated to reduce emissions markedly (38 to 6.2 tonnes PM_2.5_ per year for the power plant and a 16 % reduction in PM_10_ and 21 % reduction in NO_2_ emissions from the congestion charge) they had little impact on ambient concentrations of air pollutants.

Models suggested that pollution levels in the cities should however continue to decline through improved technology. Improved technology in this context chiefly involves firstly reduced emissions from power plants, industry and refineries and secondly the adoption of the EURO 6 vehicle emissions standards will be supported by further technological innovation to reduce transport emissions. Additionally some results appeared to imply that higher penetration of diesel cars might also reduce some harmful emissions but strict diesel emission standards would be essential because currently diesel emissions are highly harmful to health [37]. Given the often transboundary nature of the question, international cooperation is necessary to ensure that air pollution is reduced.

One policy was an exception: switching domestic heating to in-home biomass burning. This was found to substantially increase emissions of air pollutants although the growth could be limited by installing appropriate dust filters. Prototypes of filters do exist that would limit the increase to acceptable levels but market penetration will only occur if emission limits for biomass are drastically reduced. Two policies in this area could combine to provide particularly harmful effects: introducing biomass burning to a house which had been insulated giving it reduced air exchange could result in particularly high levels of indoor pollution if the burners are not completely sealed from the indoor environment.

#### Noise

Changes in exposure to noise were modelled in three of the seven cities (Additional file 1: Table A2b). Promoting electric cars and reducing use of personal cars only had a very limited effect on reducing noise levels. Similar to air pollution, the cities do not have jurisdiction over all the traffic noise: in Rotterdam, for example, 35 % of traffic is on motorways and would not be affected by local measures to reduce traffic or promote electric cars. The introduction of a metro in Thessaloniki was predicted to reduce noise levels significantly.

#### Physical activity

In Basel a large number of extra trips would be made using active transport as a result of measures to discourage car use (Additional file 1: Table A2b). Given past efforts made towards sustainable transport in Basel, however, this would only increase the share of active transport mode trips by 1 %. In Stuttgart the introduction of a congestion charge was estimated to increase active transport by a fifth regionally and 2 % in the city. Measures to increase cycling were estimated to increase the number of cycling trips also by 2 %.

#### Interim conclusions

At city level, GHG reduction policy impact on health impacting exposures was generally small at least in part as a result of previous adoption of policies. Policies with the potential to reduce air pollution significantly are generally at a national or international scale such as enforcing EURO 6 emission standards for cars and reducing industrial emissions.

### Physical health, disease and mortality

#### Impact of climate change on physical health, disease and mortality

What would the impact of climate change on physical health be if there were no GHG reduction policies? Our review of the literature [23] suggested that climate change has already adversely impacted health. In the year 2000, for instance, there were 150 000 more deaths and 5.5 million more lives affected by poor health globally than would have occurred without climate change. This toll is expected to grow from increased infectious diseases; the spread of food, water and vector borne diseases; reduced quality and availability of drinking water and deaths from flooding, fires and droughts, via food shortages and accidents, for example.

Climate change is leading to rises in average temperature globally; in China, for example, average temperature increased at a rate ranging from 0.03 °C (10 yr)^−1^ to 0.12 °C (10 yr)^−1^ over the past century [38]. There is a quadratic relationship between temperature and mortality with higher mortality risk at low or high temperatures. The case-crossover mortality study in Rotterdam [25] found significant higher mortality risks above average daily temperatures of about 26 °C or higher; thus the results support other research suggesting that heat waves increase mortality even in relatively cold regions [39]. There was also substantial interaction of temperature with air pollution, so that on warm days with increased levels of air pollution mortality risk is even higher [25]. GHG reduction policies which also reduce air pollution may therefore produce co-benefits for health.

#### Impact of GHG reduction policies on physical health, disease and mortality

Previously we saw that the GHG policies were modelled to have a small effect on reducing ambient air pollution with the exception of industry and transport emission reduction policies; additionally biomass burning was found to increase air pollution. Noise effects were again small. Physical activity had the potential to be of interest. These findings lead to similar patterns for health (Additional file 1: Table A3).

Three energy policies were considered: changing the fuel mix of local heat and power plants, encouraging in-home biomass burning for domestic heating and changing domestic heating to other lower emission fuels (gas, solar and wind power, and waste heat from industry). The power plant studied had little impact on local background levels of air pollution so health effects were negligible; in home biomass burning for domestic heating was found to increase morbidity and mortality but changing domestic heating to other lower emission fuels has potential beneficial effects.

Similarly the air pollution reduction from increasing energy efficiency of buildings is limited and consequently health effects are small. However reduced air exchange from increased energy efficiency could be detrimental to health if indoor air is polluted from tobacco smoke or an open fire.

The transport policies considered were promoting cars with lower emissions, encouraging non car modes of transport (active transport and public transport) and discouraging car use. Cars could produce lower emissions through alternative fuels such as electricity or diesel or by improved technology. Although at least small benefits for health were found for all, the largest were found for improved technology: in Rotterdam alone over 2000 lives should be saved by the adoption of EURO 6 emission standards.

Encouraging public and active transport would promote health. A new metro in Thessaloniki could reduce local deaths from air pollution by about one fifth. Preliminary analysis from Rotterdam suggested that increasing cycling could have important health benefits. However the more detailed analysis in Basel suggested that benefits were limited at a city-wide level: although benefits for individuals could be large (and outweighed the harms of breathing in higher levels of air pollution [21]), the active transport share of trips would only grow by 1 %, despite its promotion. This small increase may reflect the existing high levels of active transport engaged in in Basel – half of all households in the city do not own a car [21].

Discouraging car use, through measures such as speed restrictions and targeting traffic along main roads [21], again had small effects on mortality and morbidity. These effects occurred through reductions in air pollution and noise.

Although individual policies often produced limited positive health impacts, the combined effects of policies are greater. For example measures to reduce car use and reduce diesel emissions may reduce deaths via elemental carbon (EC) by a third. The welcome news is that policies already in place were predicted to provide large health benefits such as the adoption of the Euro 6 policies for car emissions. Thus GHG reduction policies have the potential to confer the greatest benefits in cities where few policies have previously been enacted so such policies are thus likely to have a more significant impact in Xi’an than Kuopio.

#### Interim conclusions

Climate change will have severe consequences for morbidity and mortality on a global scale. With the exception of using biomass for domestic heating, the GHG reduction policies explored were likely to improve health particularly in areas where few such policies have been adopted previously.

### Wellbeing

#### Impact of climate change on wellbeing

Our review of the literature [23] suggests that, as with health, climate change is already exerting a negative effect on wellbeing. Sudden adverse changes in the environment are associated with negative subjective feelings and emotions: floods, for example, are associated with post-traumatic stress disorder. Hotter temperatures can reduce motivation and ability to engage in physical exercise resulting in a reduction of the health and wellbeing benefits of such exercise.

#### Impact of GHG reduction policies on noise related wellbeing

Three cities considered the impact of noise changes from modelled GHG reduction policies on levels of noise annoyance during the day and sleep disturbance at night (Additional file 1: Table A4a). Rotterdam and Thessaloniki found small but positive changes from promoting electric cars, developing a new metro and reducing traffic. In Basel the results were more complex with some scenarios suggesting that noise related wellbeing would decline despite the introduction of such policies.

#### Impact of GHG reduction policies on overall wellbeing

Multivariate analysis of the cross sectional surveys suggested that wellbeing is most strongly associated with health and relationships with other people in Kuopio [30, 40] and health and youth in Suzhou [41]. Thus GHG reduction policies that improve health, such as active transport through increasing physical activity, and social capital are likely to benefit overall wellbeing [6]. Nevertheless there was some evidence that noise reduction through reduced noise annoyance can improve overall wellbeing both from extrapolation from scenarios (from promoting electric cars, underground, rail and discouraging car use) [18], and the Suzhou cross sectional survey. In Kuopio similar analysis suggested that improved thermal comfort from insulation could potentially improve overall wellbeing levels.

Other GHG reduction policies that have the potential to benefit overall wellbeing include easy access to greenspace from the home and developing high tech industry through availability of satisfying occupations. Other policies may have a neutral effect on wellbeing such as parking management or promoting alternative transport modes to the car. There are some policies that could reduce levels of wellbeing if they are not managed properly. Poor indoor air was associated with poorer wellbeing. Thus biomass burning, energy efficiency improvements with insufficient ventilation and discouraging air conditioning have potential negative consequences for wellbeing.

#### Impact of GHG reduction policies on financial wellbeing

Some cities included cost benefit analyses of their policies (Additional file 1: Table A4b). Two GHG reduction policies could have a detrimental effect: in China fast industrial growth has underpinned economic growth and cities may be concerned that slowing the growth of polluting industries may also slow economic growth [24] although opportunities for green growth do exist; secondly domestic biomass will increase costs via morbidity and mortality due to air pollution. In Stuttgart there was a negative financial impact even after taking into account benefits of CO_2_ emission reduction and the most efficient boilers. However the local topography causes particularly high local impacts [42].

In Thessaloniki all transport measures were found to be cost effective and would save more than a billion euros in health costs. In Basel a cost benefit analysis was not undertaken. However the ratio of change in DALYs to change in CO_2_ emissions was calculated and 50 % electric cars and 10 % reduction in traffic was found to be more beneficial than those measures already planned [21]. In Kuopio the GHG policies modelled did not include noise reduction as a co-benefit. However existing noise levels do cause morbidity in Kuopio so there is potential for GHG reduction measures that impact noise to have monetary benefits. In Kuopio the combined measures and in Thessaloniki building measures overall were found to be cost effective.

#### Interim conclusions

Climate change is likely to reduce wellbeing. The majority of URGENCHE analyses focussed on noise impacts on wellbeing. Reducing noise was found to positively impact noise related wellbeing and overall wellbeing. Other GHG reduction policies with impacts on overall wellbeing include easy access to green space and the replacement of high emission industries with high tech low emission industries with accompanying satisfying jobs. Policies that might increase indoor air pollution could have a negative relationship with wellbeing. Policies generally had positive economic impacts with the exception of domestic biomass burning.

### Socioeconomic inequalities

#### Impact of climate change on inequalities

Our review [23] suggested that climate change will exert more severe impacts on more disadvantaged and vulnerable populations in three ways. Firstly the negative effects of climate change will be greatest in areas predominated by already low income countries such as sub Saharan Africa. Secondly within countries, the poor will suffer more severely from adverse climactic events. Our study of heat stress and air pollution [25] supported this: mortality from hot days with high levels of air pollution was particularly high in groups with various vulnerabilities due to age, ethnicity, marital status or low income. Thirdly disadvantaged groups may be least likely to benefit from measures to adapt to climate change, for example they may be less likely to use greenspace.

#### Impact of GHG reduction policies on inequalities

Low income populations in Basel, Rotterdam and Thessaloniki experience higher noise levels and lower wellbeing. However transport policies to reduce noise did not produce particularly large improvements in wellbeing in these groups [18]. The cross sectional surveys also suggested that low socioeconomic status populations in Kuopio and Suzhou experience significantly lower wellbeing and multivariate analysis suggested that the inequality was related to comparisons with other people, exposure to stress and poor indoor air [40]. Thus interventions that have the potential to reduce indoor air quality, such as domestic biomass burning and building insulation without adequate ventilation may increase inequalities in wellbeing. A policy to reduce the use of air conditioning may reduce inequalities as air conditioning is more used by more affluent groups but may reduce wellbeing overall.

#### Interim conclusions

Low income groups and other vulnerable populations are likely to suffer disproportionately from climate change. Their needs should be taken into account when adaption and mitigation policies are being developed.

### CO_2_ emission reduction and possible co-benefits or harms for health and wellbeing

URGENCHE has explored many GHG reduction policies (Table 1) however there are four policies with analysis of scenarios by multiple cities and we can make good conclusions about their ability to reduce CO_2_ emissions and possible co-benefits or harms for health and wellbeing. The energy policy is heating though in-home biomass burning. Adopting this policy might reduce fossil CO_2_ emissions (depending on life cycle emissions of the biomass (including transport for example) which was not included in calculations) but was significantly detrimental to health in all cities where it was modelled. The buildings policy was renovation to increase energy efficiency. The benefits to CO_2_ emission reduction tended to be slight, if they existed at all, but there would be health and wellbeing co-benefits. Again these would be small. However, as with all policies, impacts are likely to be greater if implemented systematically for decades [29]. Two transport policies were studied extensively. The benefits of electric cars for reducing CO_2_ emissions are dependent upon the source of the electricity. Some health and wellbeing co-benefits are possible. Reducing car use would be likely to reduce CO_2_ emissions and sometimes had positive health impacts.

**Table 1:** Which GHG reduction policies do reduce CO_2_ and have co-benefits for health?

Policy	CO_2_	Air pollution	Health	Wellbeing	Financial wellbeing	Cities *(in brackets if policy not included in full scenario)*
*ENERGY*						
*Domestic*						
In-home biomass heating	++	--	--	-- (indoor air)	--	Stuttgart, Thessaloniki, Kuopio, Basel
Local fuel mix:oil/wood to gas/electric			++			Thessaloniki
Solar/wind	++	+	+			*(Kuopio)*
Waste heat from industry		+	+			*(Rotterdam)*
*Power generation*						
Local fuel mix: oil/wood to gas/electric			++			Thessaloniki
Local power plant: peat/oil to wood	++	+	+			Kuopio
Geothermal	Less than above					*(Kuopio)*
Improve translational efficiencies	++					*(Suzhou)*
*Industry*						
Reduce energy intense industry	Only if industry not moved elsewhere			+ (job satisfaction)	--	*(Suzhou)*
Cleaner technology		++				Rotterdam, Suzhou, Xi’an, Basel
Carbon capture and storage					--	*(Rotterdam)*
*BUILDINGS*						
Building renovation to improve energy efficiency	Sometimes outpaced by growth of new buildings	Depends on ventilation, second hand smoke (SHS), domestic heating source	Depends on ventilation, SHS, domestic heating source	Warmth helpful but depends on ventilation	++	Kuopio, Basel, Thessaloniki *(Rotterdam)*
Building isolation		+				*(Rotterdam)*
Discourage air conditioning				-		*(Suzhou)*
Increase access to greenspace				+=		*(Kuopio) (Suzhou)*
*TRANSPORT*						
*Promote cars with lower emissions*						
Increase electric cars	Depends on source of electricity	=++	++	++ noise	++	Rotterdam, Suzhou, Xi’an, Basel, Thessaloniki *(Kuopio)*
Promote diesel		++ (if emissions controlled)	++			Thessaloniki
Car emissions change to Euro 6	++ but does not reduce overall emissions due to car fleet growth	++	++			Rotterdam, Suzhou, Xi’an, Basel,
Biofuels	++ but emissions from growth/transport not calculated					Kuopio
*Encourage alternative transport models*						
Light rail more frequent throughout the day	+	+				Stuttgart
New metro	++			+noise		Thessaloniki
Improved cycling network	+	+	+	+health		Stuttgart, Basel *(Rotterdam)(Suzhou)*
*Discourage car use*						
Congestion charge	++	+				Stuttgart
Parking management	++	+		=		Stuttgart *(Kuopio)*
Traffic reduction -general	++	+	=+	=+		Rotterdam, Suzhou, Xi’an, Basel, Stuttgart

++ positive impact, + minor possible positive impact, = no impact, -minor possible negative impact, -- positive impact

In addition the effect of the already implemented policy of reducing emissions from cars to meet Euro 6 standards was studied in five cities. This is not however a city policy but one of national and regional governance. This was found to be a more effective policy for reducing CO_2_ emissions than introducing electric cars or reducing car use [15] but due to expected increases in the number of vehicles on the road, emissions would be stable in Europe and grow in China. Concurrent reductions in other air pollutants would have positive health consequences.

## Discussion

Recall URGENCHE sought to model the likely future health and wellbeing effects of energy, buildings and transport GHG reduction policies. A particular strength of the project was that city councils were embedded in the project and so the project was grounded in real city interests. One of the main findings however was that the policies the cities were interested in were not those that would have the most impact on GHG emission reduction. Three policies (renewable energy [43], carbon capture and storage [44] and reduction in heavy industry [24]) that could have major effects and cobenefits [43] were of limited interest to the cities.

A further major finding from the URGENCHE project was that cities and indeed policies should not be seen in isolation. For example reducing CO_2_ emissions from the city will not reduce CO_2_ emissions globally if industry simply moves elsewhere; several cities were interested in electric cars but for only one city would they be clearly beneficial to CO_2_ emissions reduction because that was the only city where electricity was generated from 100 % renewable sources. Thus the transport policies adopted need to take into account energy policies.

The third major finding of the project was that burning biomass is of interest to cities. Although CO_2_ emissions reduce (although this conclusion is dependent on assumptions on carbon neutrality which are less and less supported [32] and availability of locally grown biomass [32, 45]), in-home biomass burning produced harms for health and wellbeing in all cities where it was modelled. Models further suggested that the health harms of in-home biomass burning outweighed the benefits of CO_2_ emissions reduction at least in monetary terms. Controlled burning in a large plant did not have these negative consequences.

A fourth major finding was that ventilation and air exchange should be paid attention to when renovating housing in order to increase energy efficiency. Without such attention, health and wellbeing may suffer from higher actual and perceived indoor air pollution. The importance of ventilation has been confirmed in another recent study [46].

Several of the URGENCHE cities had already adopted many CO_2_ emissions reduction policies prior to the study period. Thus although we often found (with the exception of domestic biomass burning), small but positive effects on both CO_2_ emissions reduction and health and wellbeing, larger effects might be found elsewhere. As an illustration, larger positive health impacts were often found in Thessaloniki, where few policies have previously been enacted, than other European cities. Thus, with the exception of domestic biomass burning, we could generally recommend the other policies investigated.

Another positive finding was that CO_2_ emissions reduction policies already adopted are likely to have beneficial effects on CO_2_ emissions and on health and wellbeing. Such policies included adopting the Euro 6 emissions standard for cars.

### Limitations

There were a number of limitations. The main methodology of URGENCHE was to develop scenarios for individual cities assuming the adoption of a combination of CO_2_ emissions reduction policies which made sense in the local context. This approach could make extrapolating the implications of individual policies in order to inform other cities more difficult and thus there are reasonable concerns about the generalisability of our findings from the URGENCHE cities to other cities. Furthermore in most cases different analyses took place within each city. This could lead to problems when comparing results: a result deemed ‘weak’ in one city could be seen as ‘satisfactory’ in another. Secondly in order to establish theoretically maximum impacts some of the policies were rather unrealistic such as expecting 50 % cars to be electric. Thirdly modelling the future is difficult as assumptions are needed to counteract the lack of data available to quantify all necessary parameters for modelling future scenarios. Thus in the future similar studies should perhaps aim to identify policies that should be rejected through clear harms, such as in home biomass burning, rather than attempting to distinguish precise differences in positive impact of policies.

The methodologies used for analyses differed in complexity and more sophisticated techniques could have been used in some analyses: for example ageing of the population could have been taken into account when estimating future health effects. There was also a lack of availability of data particularly for some cities. For example various health-relevant indoor environmental exposures which are potentially affected by housing energy efficiency measures could have been considered (such as mould, indoor temperature or radon [46, 47]) but were not because data was not easily available or because it was not an area of expertise.

Wellbeing was mainly operationalised using the WHO-5 Wellbeing scale as it is short, the items have good face value validity, it has been successfully statistically validated, is available in many languages and was part of the EQLS survey [6]. Many alternative scales are available and WHO Europe currently recommends using items measuring satisfaction only [6]. Other academics believe that wellbeing should not be measured using a scale at all and can only be studied through qualitative methodologies such as in depth interviews [48]. Nevertheless wellbeing has been operationalised through validated scales in a substantive body of literature relating to climate change and urban policies [6, 23].

The review of wellbeing impacts of GHG policies [6] revealed major weaknesses in the literature. Due to the limited ERFs available, a novel methodology was developed [18]. However the wellbeing methodology presented here was based on cross sectional data [18, 40]. This is of particular concern for wellbeing where the direction of causality is often ambiguous. Thus all wellbeing results should be regarded as tentative.

The main focus of the wellbeing analysis via scenarios was noise. However there are many other factors that could have been included [6] such as feelings of wellbeing from physical exercise and social capital from active transport, unemployment due to changes in power generation work force and changes in access to other opportunities brought about by changing transport mode availability.

There were also other policies that could have received more focus including open green and blue space in cities and reducing housing isolation (preferring flats rather than detached houses in new builds); a recent study found that urban form may have an important role to play in urban climate change mitigation [49]. Additionally crosscutting effects of policies were not always modelled. The effect of better insulation of houses, for example, could lead also reduction of noise exposures and lead to improved health and wellbeing. Unfortunately this was not assessed in the scenarios targeted to improve energy efficiency. Biofuels replacement of food crops and negative consequences on health of populations local to where they are grown [50] were also not modelled. Furthermore implications of increasing active transport for accidents was not modelled for URGENCHE but elsewhere the extra physical activity and reduction in CO_2_ emissions have been found to outweigh any increase in accidents [51]. A systems approach has already been employed for studying housing, energy and wellbeing [52] and such an approach may also have benefits for studying the effects of climate change policies on health and wellbeing.

Enforcement of policies was not considered in the project. Policies will only realise benefits if they are enforced. In Xi’an in home and small industry coal burning was theoretically banned in 1999 [53]. However such domestic burning was still causing pollution in 2003 and 2004 [54] and in energy mix data for 2008 [17].

Some cities were more engaged with the project overall and particular parts of the project than others and this affected the scenarios modelled, for example the modelling of energy balances was limited. However in other ways this improved the breadth of the project.

## Conclusions

We are aware that policy makers at all levels of government, from cities through national to EU-wide levels, are keen to understand the practical implications of their climate change reduction policies on other policy areas, such as health. Our study considered several criteria in evaluating local mitigation policies such as health, wellbeing, inequalities, CO_2_ emissions, and economics. As we have pointed out, the impacts on some criteria could be positive whilst the impacts on other criteria could be negative. We recognise that it is not easy for policy makers to take into account conflicting impacts when evaluating mitigation policies in practise, and we thus refer policy-makers to decision-support approaches such as multi-criteria decision analysis [55] to aid their decision-making processes. In addition, the calculations for URGENCHE showed that local measures for 2020, particularly in Europe, have little additional benefit to improve air quality on top of policies already implemented (the low-hanging fruit have already been picked), and when compared to other larger national policies.

Notwithstanding these issues of conflicting impacts discussed above and that our results are based on seven cities and extrapolation from these to other cities should be undertaken with caution, we summarise the following main policy implications arising from the URGENCHE findings:

- Overarching impacts
  - Given that some of the largest predicted impacts on CO_2_ emissions were from new technologies which would allow vehicles to meet Euro 6 standards and reduce industrial emissions, international bodies and national governments need to encourage the development of new technologies to reduce emissions and legislate for their uptake.
  - When considering the adoption of a GHG reduction policy, city planners need to take into account proximal and distal impacts.
  - Economic and population growth, particularly in China, will annul small GHG reduction impacts of less ambitious policies.
- Transport related
  - Electric cars may confer some small health benefit but it would be unwise for a city to invest unless their power generation fuel mix is less polluting than vehicle fuels (petrol (gasoline) and diesel).
  - Reducing GHG emissions through discouraging private car use and promoting and supporting public and active transport modes incurs health co-benefits chiefly by reducing noise and increasing physical activity. However reductions in air pollution were less apparent due to concomitant growth in the vehicle fleet and motorway traffic which was not subjected to local traffic reduction measures. Thus local policies may have health co-benefits but national policies are also needed.
- Building related
  - Renovation of buildings can exhibit positive or negative health and wellbeing impacts often due to insufficient attention paid to ventilation - irrespective of the energy conserved. This highlights the need for epidemiological research on renovation as well as training and auditing of renovation engineers, supervisors and labourers.
  - More data collection on indoor levels of damp and thermal comfort would aid assessment of the health effects of energy efficient housing
- Energy related
  - Cities could make use of the URGENCHE model to analyse their energy policies. As long as the city has recorded a full year’s ‘energy balance’ (describing import and flow of energy, energy conversion and use of energy sources within a city) the model can be used to assess the potential impact of different energy policies on emissions of GHG and air pollutants.
  - New technologies for reducing in home biomass burning emissions are required.
  - The creation of a European Energy Union [36] which is intended to enable transfer of surplus renewably generated energy would allow cities with an energy mix based on greenhouse gas neutral renewables to export energy and would motivate further energy saving measures in these cities.

## Additional files

Additional file 1:
**Results tables.** (DOCX 175 kb) Additional file 2:
**Peer review reports.** (PDF 435 kb)
