# Supplementary material for: Public health impacts of city policies to reduce climate change: findings from the URGENCHE EU-China project
Source: Environ Health. 2016 Mar 8;15(Suppl 1):25. doi: 10.1186/s12940-016-0097-0 (PMC4895602; doi:10.1186/s12940-016-0097-0)
Supplement: Additional file 1: — Results tables. (DOCX 175 kb) [file 12940_2016_97_MOESM1_ESM.docx]

# Additional file 1 for Sabel et al Public Health impacts of city policies to reduce climate change: findings from the URGENCHE EU-China project

Note that this file has its own reference list so the reference numbering is different to that of the main article

# Results tables

Box A1 Key to additional tables

|  |  |  |  |  |  |
| --- | --- | --- | --- | --- | --- |
|  |  | *Impact of policy* |  |  |  |
|  |  | ++ |  | The policy had a sizeable positive impact (CO_2_ emissions declined or health and wellbeing likely to be improved) |  |
|  |  | + |  | The policy may have had a positive impact but the authors suggested it was small |  |
|  |  | = |  | The policy had no impact |  |
|  |  | - |  | The policy may have had a negative impact but the authors suggested it was small |  |
|  |  | -- |  | The policy had a sizeable negative impact (CO_2_ emissions increased or health and wellbeing likely to be worse) |  |
|  |  | ? |  | Unknown or ambiguous |  |
|  |  |  |  |  |  |
|  |  | *Source of evidence* |  |  |  |
|  |  |  |  | Policy analysed through a full scenario approach |  |
|  |  |  |  |  |  |
|  |  |  |  | Policy analysed using a supplementary scenario |  |
|  |  |  |  |  |  |
|  |  |  |  | Policy analysed through a longitudinal study |  |
|  |  |  |  |  |  |
|  |  |  |  | Result of policy implied through an association with wellbeing found in multivariate analysis of the Kuopio or Suzhou cross sectional wellbeing surveys |  |
|  |  |  |  |  |  |
|  |  |  |  | Other e.g. preliminary analysis only |  |
|  |  |  |  |  |  |
|  |  | *Abbreviations* |  |  |  |
|  |  | /a |  | Per year |  |
|  |  | t/a |  | Tons per year |  |
|  |  | ↑ |  | increase |  |
|  |  | ↓ |  | decrease |  |
|  |  | Lden |  | Equivalent continuous noise level over 24 hours (noise in the evening and night is weighted higher) |  |
|  |  | Lnight |  | Equivalent continuous noise level during the night (23:00 to 07:00) |  |
|  |  | DBA |  | Decibel (unit of sound pressure) |  |
|  |  | DALY |  | a measure of overall disease burden, expressed as the number of years lost due to death (YLL) added to the number of years lived with a disability or illness (YLD) |  |
|  |  | C_6_H_6_ |  | Benzene |  |
|  |  | CO_2_ |  | Carbon dioxide |  |
|  |  | EC |  | Elemental carbon (also known as black carbon) |  |
|  |  | NH_3_ |  | Ammonia |  |
|  |  | NO_x_ NO_2_ |  | Nitrogen oxides Nitrogen dioxide |  |
|  |  | PM_2.5_ PM_10_ |  | Particulate matter (up to 2.5 or 10 micrometres) |  |
|  |  | SO_2_ |  | Sulphur dioxide |  |
|  |  | UI |  | Uncertainty Interval |  |
|  |  |  |  |  |  |

Table A1a Effect of buildings and energy policies on CO_2_ emissions

| Policy | City | Change in CO_2_ emissions | Effect of policy on local CO_2_ emissions | Effect on global  CO_2_ emissions | Ref |
| --- | --- | --- | --- | --- | --- |
| *ENERGY* |  |  |  |  |  |
| *Domestic heating*^#^ |  |  |  |  |  |
| Pellet boilers not banned | Stuttgart | CO_2_ emissions decline of 65.5 kt or 10.6% but emissions from making/transporting pellets not calculated | ++ | ? | [1] |
| Wood burning not discouraged | Kuopio | CO_2_ emissions decline of 7020 tons/a or 1% | + | + | [2] |
| Post 2010 wood & oil and 50% existing electric powered residences powered by solar/wind | Kuopio | CO_2_ emissions decline of 18070 tons/a | ++ | ++ | [2] |
| *Electricity generation* |  |  |  |  |  |
| 84% peat, 12% heavy oil & 4% wood changed to 49% peat 1% heavy oil & 50% wood^#^ | Kuopio | CO_2_ emissions decline of 297403 tons/a or 45%^#^ | ++ | ++ | [2] |
| Geothermal | Kuopio | Less reduction than changing the power plant from peat to wood due to electricity needed for generation | ++ | ? | [2] |
| *Industry* |  |  |  |  |  |
| Reduce energy intense industry | Suzhou | Carbon intensity decreased from 1.05 to 0.73 tCO2e/KUS$^*^ from 2005 to 2010. Some decline through technological innovation but some from displacement of heavy industry to other cities. | ++ | ? | [3] |
| Improve translational efficiencies | Suzhou | Big contributions to energy saving and emissions reduction | ++ |  | [4] |
|  |  |  |  |  |  |
| BUILDINGS |  |  |  |  |  |
| sheath reform renovation leading to heat energy need reduced to 25 kWh/m2 | Kuopio | CO_2_ emissions decline of 2876 tons/a but subsequent CO2 emission reduction offset by increase in building stock | ++ | = | [5] [2] |
| 2% pre 1980 buildings renovated pa (vs 1%) | Basel | “Impact” but offset by increase in building stock | ++ | = | [5] |
| 100% pre 1980 buildings renovated | Basel | “Significantly reduced” | ++ | ++ | [5] |

^#^The power plant modelled is used for producing district heating in addition to producing power *Thousands of US Dollars

Table A1b Effect of transport policies on CO_2_ emissions

| Policy | City | Change in CO_2_ emissions | Effect of policy on local CO_2_ emissions | Effect on global  CO_2_ emissions | Ref |
| --- | --- | --- | --- | --- | --- |
| *Promote cars with lower emissions* |  |  |  |  |  |
| 50% cars electric | Basel | 19% emission reduction – All electricity is from renewable sources in Basel | ++ | ++ | [6] |
| 50% cars electric | Rotterdam Suzhou Xian (Basel) | 25% city emission reduction but electricity generation emissions would increase (except for Basel) | ++ | ? | [7] |
| 10% cars & all buses electric | Kuopio | CO_2_ emissions from transport decline by 7906 tons/a but the local power plant produces CO_2_ emissions. | ++ | ? | [2] [5] |
| The average car emission changes from Euro 4 to 5 in 2010 to Euro 5 to 6 in 2020 | Rotterdam, Basel | In 2010/2020, CO_2_ emissions in grams per vehicle kilometre: 306/304 (Basel), 230/215 (Rotterdam). Due to fleet increases overall emissions will be similar. | ++ | = | [7] |
| The average car emission changes from Euro 3 in 2010 to Euro 5 to 6 in 2020 | Suzhou Xian | In 2010/2020, CO_2_ emissions in g per vkm: 420/394 (Xi'an) and 380/378 (Suzhou). Due to fleet growth overall emissions will increase. | ++ | - | [7] |
| 30% vehicle fuel is biofuels (vs 20%) and car emissions change from EURO 4 to 5 | Kuopio | CO_2_ emissions decline of 11445 tons/a but emissions from growing/transporting biofuels not calculated | ++ | ? | [2] |
| *Encourage alternative transport modes* |  |  |  |  |  |
| light rail more frequent throughout the day | Stuttgart | CO_2_ emissions decline by 0.1% | + | + | [8] |
| New metro | Thessaloniki | CO_2_ emissions decline by 22% to 33% near metro | ++ | ++ | [9] |
| improved cycling network | Stuttgart | CO_2_ emissions decline by 0.1% | + | + | [8] |
| *Discourage car use* |  |  |  |  |  |
| Congestion charge/toll | Stuttgart | CO_2_ emissions decline by 14.0% | ++ | ++ | [8] |
| parking management | Stuttgart | CO_2_ emissions decline by 1.8% | ++ | ++ | [8] |
| All measures (light rail,  cycle network, toll, parking) | Stuttgart | CO_2_ emissions decline by 15.6% | ++ | ++ | [8] |
| 4% reduction of personal cars | Basel | CO_2_ emissions decline by 1% from 2010 | + | + | [6] |
| 10% reduction of personal cars | Rotterdam Suzhou Xian Basel | CO_2_ emissions decline by 5% | ++ | ++ | [7] |

Table A2a Effect of policies on health impacting exposures: air pollution

| Policy | City | Effect of policy on PM_2.5_ emissions | | | Effect of policy on PM_10_ emissions | | | Effect of policy on other pollutants | | | | | Ref |
| --- | --- | --- | --- | --- | --- | --- | --- | --- | --- | --- | --- | --- | --- |
|  |  | Emitted^1^ | Exposure^2^ | Sign^3^ | Emitted^1^ | Exposure^2^ | Sign^3^ | Pollutant | Emitted^1^ | | Exposure^2^ | Sign^3^ |  |
| *ENERGY* |  |  |  |  |  |  |  |  |  | |  |  |  |
| *Electricity generation* |  |  |  |  |  |  |  |  |  | |  |  |  |
| 84% peat, 12% heavy oil & 4% wood changed to 49% peat 1% heavy oil & 50% wood^7^ | Kuopio | 38 to 6.2 t/a,  .05% share | .003-.001  c: μg/m3 | + | 54 to 11 t/a |  | + | NO_x_  SO_2_ | 65% ↓  79%↓ | |  | +  + | [2] |
| Post 2010 wood & oil and 50% existing electric powered residences solar/wind powered | Kuopio | 0.25 to 0 μg/m^3^ |  | + |  |  |  |  |  | |  |  | [2] |
| Residences powered by waste heat from industry | Rotterdam | ‘Not great’^5^ |  | + |  |  |  |  |  | |  |  | [8] |
|  |  |  |  |  |  |  |  |  |  | |  |  |  |
| *Domestic heating* |  |  |  |  |  |  |  |  |  | |  |  |  |
| In home wood burning not discouraged | Kuopio | 9 to 24 t/a, 4% share | .20 to .32  c: μg/m3 | - | 10 to 26 t/a |  | - |  |  | |  |  | [2] |
| Electric fuel & gas taxation –biomass increases 5.6 to 20.7% | Thessaloniki |  | 34.6-42.7  pw: μg/m3 | -- |  | 39.6-47  pw: μg/m^3^ | -- |  |  | |  |  | [10] |
| Electric fuel & gas taxation –oil ↓ 38.5% -23,5%, gas↑ 41.5%-66.5% | Thessaloniki |  | 40.8 -19.9  μg/m3 | -- |  |  | -- |  |  | |  |  | [11] |
| Pellet boilers not discouraged | Stuttgart | 17,1 t^4^ |  | -- | 19,5 t^4^ |  | *--* |  |  | |  |  | [1] |
|  |  |  |  |  |  |  |  |  |  | |  |  |  |
| *Industry* |  |  |  |  |  |  |  |  |  | |  |  |  |
| Cleaner technology in all polluting sectors (industry, power plants, agriculture) (vs 2010) | Rotterdam Suzhou Xian Basel | ↓ | ↓ | ++ | ↓ | ↓ | ++ | NO_x_  SO_2_  NH_3_ | ↓  ↓  ↓ | | ↓  ↓  ↓ | ++  ++  ++ | [12] [7] |
|  |  |  |  |  |  |  |  |  |  | |  |  |  |
|  |  |  |  |  |  |  |  |  |  | |  |  |  |
| *BUILDINGS* |  |  |  |  |  |  |  |  |  | |  |  |  |
| sheath reform renovation leading to heat energy need reduced to 25 kWh/m^2^ | Kuopio | 10 to 9 t/a |  | + | 11 to 10 t/a |  | + |  |  | |  |  | [2] |
| Housing insulation | Rotterdam | ‘Not great’^5^ |  | + |  |  |  |  |  | |  |  | [8] |
| Housing isolation | Rotterdam | “Small”^5^ |  |  |  |  |  |  |  | |  |  | [13] |
| Energy Efficiency Certification | Thessaloniki |  | ↑ indoor  ↓outdoor  29.5-23.3  μg/m^3^ | ? ^6^ |  |  |  |  |  | |  |  | [11] |
|  |  |  |  |  |  |  |  |  |  | |  |  |  |
| *TRANSPORT* |  |  |  |  |  |  |  |  |  | |  |  |  |
| *Promote cars with lower emissions* |  |  |  |  |  | |  |  |  | |  |  |  |
| 50% cars electric | Rotterdam Suzhou Xian Basel |  |  | = |  |  | = | NO_2_  EC | |  | =  = | =  = | [7] |
| 50% cars electric | Rotterdam |  |  | = |  |  |  | EC  background | | ↓ | .5 to .4  p:μg/m^3^ | + | [12] |
| 22% cars diesel | Thessaloniki |  |  |  |  |  |  | NO_2_ C_6_H_6_ | |  |  | ++ | [11] |
| 50% cars electric | Thessaloniki |  |  |  |  |  |  | Air pollutants | |  |  | ++ |  |
| 10% cars & all buses electric | Kuopio | “Minor” | “Minor” | + |  |  |  |  | |  |  |  | [2] |
| 30% vehicle fuel is biofuels (vs 20%) and car emissions change from EURO 4 to 5 | Kuopio | 33 to 20 t/a, 7% share | .35 to .21  c: μg/m^3^ | + | 33 to 20 t/a | + |  |  | |  |  |  | [2] |
| Car emissions change to EURO 6 | Rotterdam Suzhou Xian Basel | ↓ | ↓ | ++ | ↓ | ↓ | ++ | EC  NO_2_ | | ↓  ↓ | ↓  ↓ | ++  ++ | [12]  [7] |
|  |  |  |  |  |  |  |  |  | |  |  |  |  |
|  |  |  |  |  |  |  |  |  | |  |  |  |  |
|  |  |  |  |  |  |  |  |  | |  |  |  |  |
| *Promote alternative transport modes* |  |  |  |  |  |  |  |  | |  |  |  |  |
| Light rail more frequent throughout the day | Stuttgart |  |  |  | 0.1%↓ | <1%↓ | + | NO_2_  NO_x_ | | 0.1%↓  0.1%↓ | <1%↓  <1%↓ | +  + | [8] [4] |
| Improved cycling network | Stuttgart |  |  |  | 0.2%↓ | <1%↓ | + | NO_2_  NO_x_ | | 0.3%↓  0.3%↓ | <1%↓  <1%↓ | +  + | [8] [4] |
|  |  |  |  |  |  |  |  |  | |  |  |  |  |
| *Discourage car use* |  |  |  |  |  |  |  |  | |  |  |  |  |
| Congestion charge/toll | Stuttgart |  |  |  | 16.2%↓ | <1%↓ | + | NO_2_  NO_x_ | | 21.1%↓  18.4%↓ | <1%↓  <1%↓ | +  + | [8] [4] |
| Parking management | Stuttgart |  |  |  | 2.2%↓ | <1%↓ | + | NO_2_  NO_x_ | | 2.2%↓  2.2%↓ | <1%↓  <1%↓ | +  + | [8] [4] |
| All measures (light rail,  cycle network, toll, parking) | Stuttgart |  |  |  | 18.1%↓ | <1%↓ | + | NO_2_  NO_x_ | | 22.7%↓  20.2%↓ | <1%↓  <1%↓ | +  + | [8] [4] |
| 4% reduction of personal cars | Basel |  | = | = |  |  |  | EC | |  | .53-.52  c: μg/m^3^ | + | [6] |
| 10% reduction of personal cars | Rotterdam Suzhou Xian Basel |  |  | + |  |  | + | NO_2_  EC | |  | +  + | + | [7] |
|  |  |  |  |  |  |  |  |  | |  |  |  |  |
| *Multiple* |  |  |  |  |  |  |  |  | |  |  |  |  |
| Agreed measures & strict diesel emission standards (vs 2010) | Basel |  | 15.05 to 9.4, 38%↓  c: μg/m^3^ | ++ |  |  |  | EC | |  | 1.58 -.53 66%↓  c: μg/m^3^ | ++ | [6] |
|  |  |  |  |  |  |  |  |  | |  |  |  |  |

^1^t/a tonnes per year; share refers to source as a share of all sources of pollutant

^2^c: city exposure, p: personal exposure, pw: personal exposure in winter ↓decline in pollutant, ↑ increase in pollutant

^3^+ beneficial decline in pollution, + possible minor decline in pollution, = no change in pollution, - possible minor detrimental increase in pollution, - detrimental increase in pollution

^4^20 t/a is for an ordinary boiler but emissions can be up to five times lower if a filter is used

^5^Little change in emissions because most dwellings in Rotterdam heated by gas

^6^Reduced air exchange will reduce outdoor pollution but increase indoor pollution if there tobacco smoke or use of an open fire in the home

^7^The power plant modelled is used for producing district heating in addition to producing power

Table A2b Effect of transport policies on health impacting exposures: noise and physical activity

| Policy | City | Change | Sign | Ref |
| --- | --- | --- | --- | --- |
| *NOISE* |  |  |  |  |
| *Promote cars with lower emissions* |  |  |  |  |
| 50% cars electric | Basel | Lden(DBA) 52.12 to 49.43 Lnight(DBA) 41.83 to 39.73 | + | [6] |
| 50% cars electric | Rotterdam | % in lowest noise exposure level(49.5 -54.5 decibels) Lden 38.2% to 39% Lnight 62.4% to 64.2% | + | [12] |
|  |  |  |  |  |
| *Promote alternative transport modes* |  |  |  |  |
| New metro | Thessaloniki | Localised impact on noise | ++ | [11] |
|  |  |  |  |  |
| *Discourage car use* |  |  |  |  |
| 4% reduction of personal cars | Basel | Lden(DBA) 52.12 to 52.01 Lnight(DBA) 41.83 to 41.80 | + | [6] |
| 10% reduction of personal cars | Rotterdam | % in lowest noise exposure level (49.5 -54.5 decibels) Lden 38.2% to 38.7% Lnight 62.4% to 64.5% | + | [12] |
|  |  |  |  |  |
| *PHYSICAL ACTIVITY* |  |  |  |  |
| *Promote alternative transport modes* |  |  |  |  |
| Light rail more frequent throughout the day | Stuttgart | No change in active transport | = | [4] |
| Improved cycling network | Stuttgart | 2% increase in number of cycling trips | + | [4] |
|  |  |  |  |  |
| *Discourage car use* |  |  |  |  |
| 4% reduction of personal cars | Basel | 7,222 extra cycling trips (calculated as 108,049-100,827 trips)  19,778 extra walking trips (calculated as 291,068-271,290).  2% more cyclists and 10% more walkers  This means a 7 percentage point increase in trips or an increase in the share of active trips of 1% | ++ | [6] [8] |
| Congestion charge/toll | Stuttgart | Walking: 10% growth regionally and 2% in the city,  Cycling: 12% growth regionally and 2% in the city | ++ | [4] |
| Parking management | Stuttgart | Negligible change in active transport | = | [4] |

Table A3 Effect of policies on morbidity and mortality

| Policy | City | Reason for change in health | Health measure | Change | Sign | Ref |
| --- | --- | --- | --- | --- | --- | --- |
| *ENERGY* |  |  |  |  |  |  |
| *Electricity & heat generation* |  |  |  |  |  |  |
| 84% peat, 12% heavy oil & 4% wood changed to 49% peat 1% heavy oil & 50% wood | Kuopio | PM_2.5_↓ | Mortality | ↓ <1 DALY/a | + | [5] |
|  |  |  |  |  |  |  |
| *Domestic heating* |  |  |  |  |  |  |
| In home wood burning not discouraged | Kuopio | PM_2.5_ ↑ | Mortality | ↑ 8 DALY/100000 | - | [2] |
| In home wood burning not discouraged | Kuopio | PM_2.5_ ↑ | Infant mortality | ↑1 DALY/100000 | - | [2] |
| In home wood burning not discouraged | Kuopio | PM_2.5_ ↑ | Chronic Bronchitis | ↑2 DALY/100000, ↑ 1 cases/a | - | [2] |
| In home wood burning not discouraged | Kuopio | PM_2.5_ ↑ | Work loss days (day when a worker is too ill to go to work.) | ↑ 171 cases/a | - | [2] |
| In home wood burning not discouraged | Kuopio | PM_2.5_ ↑ | Restricted activity days (a day where a person cannot do all normal activities they normally do (or they have to increase medication to compensate for the symptoms), but without the need for a sick leave | ↑ 18 cases/a | - | [2] |
| In home wood burning not discouraged | Kuopio | PM_2.5_ ↑ | Hospital admissions (CVD, respiratory) (a situation where a person is either treated in the hospital or stays in the hospital for observation – not just a visit to a hospital doctor) | 0 DALY/100000, 0 cases/a | = | [2] |
| Pellet boilers not banned | Stuttgart | PM_2.5_ ↑ PM10↑ | Mortality, morbidity | ↑ between 10.1 and 69.4 DALY/a depending on whether the boiler is fitted with a filter and/or is low emission | -- | [1] |
| District heating changed from 40% gas to 20% gas and 20% wood | Basel | PM_2.5_ ↑ | Mortality | ↑ 11 DALY | - | [5] |
| Electric fuel & gas taxation leading to biomass burning | Thessaloniki | PM_2.5_ ↑ | Mortality, morbidity | ↑ 200 deaths/a in a population of 900 000 | -- | [8] |
|  |  |  |  |  |  |  |
| Post 2010 wood & oil and 50% existing electric powered residences become solar/wind powered | Kuopio | PM_2.5_↓ | Mortality | “health benefit would be negligible” | + | [2] |
| Electric fuel & gas taxation –oil ↓ 38.5% -23,5%, gas↑ 41.5%-66.5% | Thessaloniki | PM_2.5_ ↓ | Mortality & infant mortality | ↓400 deaths/a in a population of 900 000 | ++ | [8] |
| Waste heat from industry to all new houses | Rotterdam | Air quality “impact small” | health | no significant change | + | [13] |
|  |  |  |  |  |  |  |
| *BUILDINGS* |  |  |  |  |  |  |
| Energy efficiency and building isolation | Rotterdam | Air quality “impact small” | health | no significant change | + | [13] |
| Sheath reform renovation leading to heat energy need reduced to 25 kWh/m^2^ | Kuopio | PM_2.5_↓ | Mortality | ↓ 5.5 DALY/a | + | [5] |
| More buildings renovated (4.5 vs 3% pa) but less efficiently | Kuopio | PM_2.5_↓ | Mortality | ↓ 3 DALY/a | + | [5] |
| 2% pre 1980 buildings renovated pa (vs 1%) | Basel | PM_2.5_↓ | Mortality | ↓ 5 DALY/a | + | [5] |
| All pre 1980 buildings renovated pa (vs 1%) | Basel | PM_2.5_↓ | Mortality | ↓ 21 DALY/a | + | [5] |
| Energy Efficiency Certification | Thessaloniki | PM_2.5_ varies | Mortality, infant mortality, hospital admissions for CVD & lung cancer | ↓ but ↑ if there is tobacco smoke or an open fire. However confidence intervals overlap. | ? | [11] |
|  |  |  |  |  |  |  |
| *TRANSPORT* |  |  |  |  |  |  |
| *Promote cars with lower emissions* |  |  |  |  |  |  |
| 50% cars electric | Thessaloniki | PM_10_↓ | mortality | ↓4% cases, 152 DALY | ++ | [8] |
| 50% cars electric | Thessaloniki | PM_2.5_↓ | mortality | ↓4% cases, 83 DALY | ++ | [8] |
| 50% cars electric | Thessaloniki | NO_2_↓ | mortality | ↓2% cases, 75 DALY | ++ | [8] |
| 50% cars electric | Thessaloniki | C_2_ H _6_↓ | Life time cases of leukaemia | ↓21% cases, 2.5 DALY | ++ | [8] |
| 50% cars electric | Thessaloniki | noise | Myocardial infarction | ↓49% cases | ++ | [8] |
|  |  |  |  |  |  |  |
| 50% cars electric | Basel | Air pollution ↓ | Mortality & Restricted activity days | 0 DALY/1000 | = | [6] |
| 50% cars electric | Basel | EC↓ | Cancer Risk | Decreased by “small amount” | + | [8] |
| 50% cars electric | Basel | Noise↓ | Mortality, annoyance, sleep disturbance | .54 DALY/1000 (1% deaths postponed) | + | [6] |
| 50% cars electric | Basel | Noise↓ | Mortality, annoyance, sleep disturbance | .54 DALY/1000 (1% deaths postponed) | + | [6] |
| Encourage diesel fuel | Thessaloniki | NO2↓ | Mortality | ↓ up to 19% | ++ | [11] |
| Encourage diesel fuel | Thessaloniki | C_6_H_6_↓ | morbidity | ↓ up to 16% | ++ | [11] |
| Car emissions not to exceed EURO6  (also included some growth in traffic) vs 2010 | Rotterdam | PM2.5↓7.41 μg/m^3^ | Mortality | 5.8 YLL/ 1,000 adults life years lost can be prevented or 2,097 (Uncertainty Interval (UI): 1,403, 2,711) life years can be saved | ++ | [12] |
|  |  |  |  |  |  |  |
| *Encourage alternative transport modes* |  |  |  |  |  |  |
| Increase in the share of active trips of 1% | Basel | Physical activity | Mortality | ↓.06 DALY/1000 (vs 2010) .03% premature deaths (<1 natural death in commuters) per year prevented | + | [8] |
| Increase cycling 30% | Rotterdam | Physical activity | health | “significant health impact” | ++ | [13] |
| New metro | Thessaloniki | PM_2.5_↓ | mortality | ↓17% | ++ | [11] |
| New metro | Thessaloniki | NO_2_↓ | mortality | ↓24% | ++ | [11] |
|  |  |  |  |  |  |  |
| *Discourage car use* |  |  |  |  |  |  |
| 4% reduction of personal cars | Basel | Air pollution↓ | Mortality | ↓.1 DALY/1000 | + | [6] |
| 4% reduction of personal cars | Basel | Noise ↓ | Mortality, annoyance, sleep disturbance | ↓.07 DALY/1000 | + | [6] |
| 4% reduction of personal cars | Basel | EC↓ | Cancer Risk | Decreased by small amount | + | [8] |
| 10% reduction of personal cars | Basel | EC↓ | Cancer Risk | Decreased by small amount | + | [8] |
| 10% reduction of personal cars | Rotterdam | Air pollution = |  | 0 | = | [12] |
|  |  |  |  |  |  |  |
| *Multiple measures -transport* |  |  |  |  |  |  |
| (unspecified) | Xian | PM_2.5_↓ 78 to 50μg/m^3^ | Mortality | ↓90 deaths (249 to 159) | + | [8] |
| Agreed traffic reduction measures & strict diesel emission standards (vs 2010) | Basel | Air pollution↓ | Mortality | ↓3.8 DALY/1000 6% of total /a | ++ | [8] |
| Agreed traffic reduction measures & strict diesel emission standards (vs 2010) | Basel | EC↓ | Cancer Risk | ↓1/3 | ++ | [8] |
| New metro and increase in cars fuelled by diesel | Thessaloniki | PM_10_↓ | mortality | ↓137 cases or 17 DALY | ++ | [8] |
| New metro and increase in cars fuelled by diesel | Thessaloniki | PM_2.5_↓ | mortality | ↓10 cases or 13 DALY | ++ | [8] |
| New metro and increase in cars fuelled by diesel | Thessaloniki | NO_2_↓ | mortality | ↓64 cases or 9 DALY | ++ | [8] |
| New metro and increase in cars fuelled by diesel | Thessaloniki | C_6_H_6_↓ | Life time cases of leukaemia | ↓.16 cases or 2.4 DALY (22%) | ++ | [8] |
| New metro and increase in cars fuelled by diesel | Thessaloniki | Noise | Myocardial infarction | ↓29% cases | ++ | [8] |
| Measures to reduce noise | Kuopio | Noise | Disease | ↓ disease by up to 33% | ++ | [2] |
|  |  |  |  |  |  |  |
|  |  |  |  |  |  |  |
|  |  |  |  |  |  |  |
|  |  |  |  |  |  |  |
|  |  |  |  |  |  |  |
|  |  |  |  |  |  |  |
|  |  |  |  |  |  |  |
| *Multiple measures -any* |  |  |  |  |  |  |
| (Unspecified) | Xian | PM_2.5_↓ 78 to 50μg/m^3^ | Mortality | ↓308 deaths (857 to 549) | ++ | [8] |
| Heat & Powerplant fuel peat & oil changed to wood, biofuels for transport, building renovation | Kuopio | PM_2.5_↓ | mortality | 2 DALY/100000 (2 deaths per year), 1 case/a | + | [2] |
| As above | Kuopio | PM_2.5_↓* | Work loss days | 64 cases/a | + | [2] |
| As above | Kuopio | PM_2.5_↓** | Restricted activity days | 6 cases/a | + | [2] |
| As above | Kuopio | PM_2.5_↓ | Infant mortality | 0 DALY/100000, 0 cases/a | = | [2] |
| As above | Kuopio | PM_2.5_↓ | Chronic bronchitis | ↓ 1 DALY/100000, | + | [2] |
| As above | Kuopio | PM_2.5_↓ | Hospital admissions CVD or respiratory | 0 DALY/100000, 0 cases/a | = | [2] |

+ health improvement, + possible minor health improvement, = no change in health, - possible minor health deterioration, - health deterioration

*ERF expressed as a relative risk (RR) RR=1,046 / 10 µg/m^3^ PM_2.5_

**ERF expressed as a relative risk (RR) RR=1,048 / 10 µg/m^3^ PM_2.5_

Table A4a Effect of energy and buildings policies on wellbeing

| Policy | City | Reason for change in wellbeing | Wellbeing measure | Change | Sign | Ref |
| --- | --- | --- | --- | --- | --- | --- |
| ENERGY |  |  |  |  |  |  |
| Domestic heating by in home biomass | Kuopio | Smoke not a problem at home | WHO-5 Wellbeing index | ↑ 13.7 (5.8 to 18.5) percentage points | - | [5] |
| Replacing high emission industry with highly technical industries | Suzhou | Satisfaction with job | WHO-5 Wellbeing index | ↑ 2.2 (1.0 to 3.5) percentage points | + | [14] |
| Discourage air conditioning | Suzhou | No fan in kitchen | WHO-5 Wellbeing index | ↓4.6 (0.9 to 8.3) percentage points | - | [14] |
| BUILDINGS |  |  |  |  |  |  |
| 3% buildings renovated/a (vs 2010) | Kuopio | Energy efficiency | Thermal comfort | 0.8 percentage point ↓ in population modelled to report cold problems | + | [8] |
| Sheath reform renovation leading to heat energy need reduced to 25 kWh/m^2^ | Kuopio | Energy efficiency | Thermal comfort | 0.7 percentage point ↓ in population modelled to report cold problems | + | [8] |
| More buildings renovated (4.5 vs 3% pa) but less efficiently | Kuopio | Energy efficiency | Thermal comfort | 0.3 percentage point ↓ in population modelled to report cold problems | + | [8] |
| 3% buildings renovated/a (vs 2010) | Kuopio | Thermal comfort | WHO-5 Wellbeing index | Likelihood of population reporting good wellbeing unchanged | = | [8] |
| Sheath reform renovation leading to heat energy need reduced to 25 kWh/m^2^ | Kuopio | Thermal comfort | WHO-5 Wellbeing index | 0.1 percentage point ↑ in population likely to be in good wellbeing | + | [8] |
| More buildings renovated (4.5 vs 3% pa) but less efficiently | Kuopio | Thermal comfort | WHO-5 Wellbeing index | Likelihood of population reporting good wellbeing unchanged | = | [8] |
|  |  |  |  |  |  |  |
| Insulation | Kuopio | Home too cold *less* of the time | WHO-5 Wellbeing index | ↑ 13.5 (9.6 to 16.6) percentage points | + | [15] |
| Insulation | Kuopio | Still air or stuffiness | WHO-5 Wellbeing index | ↓ 12.1 (4.8 to 16.4)percentage points | + | [15] |
|  |  |  |  |  |  |  |
| Increase greenspace | Kuopio | Satisfied with distance to greenspace | WHO-5 Wellbeing index | ↑ 13.2 (7.7 to 17.0) percentage points | + | [15] |
| Increase greenspace | Kuopio | Spends free time in nature more often | WHO-5 Wellbeing index | ↑ 13.8 (10.3 to 16.6) percentage points | + | [15] |
| Increase greenspace | Suzhou | Time taken to access greenspace | WHO-5 Wellbeing index | Non significant relationship | = | [14] |

+ wellbeing improvement, + possible minor wellbeing improvement, = no change in wellbeing, - possible minor wellbeing deterioration, - wellbeing deterioration

Table A4b Effect of transport policies on wellbeing

| Policy | City | Reason for change in wellbeing | Wellbeing measure | Change | Sign | Ref |
| --- | --- | --- | --- | --- | --- | --- |
| *Promote cars with lower emissions* |  |  |  |  |  |  |
| 50% electric cars | Basel | Noise ↓ | Noise annoyance | .37↓ DALY /1000 | - | [6] |
| 50% electric cars | Basel | Noise ↓ | Sleep disturbance | .01↑ DALY /1000 | - | [6] |
| 50% cars electric | Rotterdam | Noise ↓ | Noise annoyance | ↓26 years lost to disability (YLD) | + | [12] |
| 50% cars electric | Rotterdam | Noise ↓ | Sleep disturbance | ↓42 (UI 23-60) YLD | + | [12] |
| 50% cars electric | Rotterdam | Noise annoyance ↓ | WHO_5 Wellbeing Index | probability of good wellbeing unchanged for city population  .7%↑ probability of good wellbeing for highly annoyed population | + | [12] |
| 50% cars electric | Thessaloniki | Noise ↓ | Noise annoyance | ↓50% | ++ | [8] |
| 50% cars electric | Thessaloniki | Noise ↓ | Sleep disturbance | ↓80% | ++ | [8] |
|  |  |  |  |  |  |  |
| *Encourage alternative transport modes* |  |  |  |  |  |  |
| New metro | Thessaloniki | Noise ↓ | Noise annoyance | ↓2% | + | [8] |
| New metro | Thessaloniki | Noise ↓ | Sleep disturbance | ↓34% | + | [8] |
| New metro | Thessaloniki | Noise annoyance↓ | WHO_5 Wellbeing Index | .5%↑ probability of good wellbeing for city population  3.7%↑ probability of good wellbeing for highly annoyed population | + | [16] |
| Change transport mode | Kuopio | Not using car | WHO_5 Index | Not significant predictor of wellbeing in multivariate analysis | = | [15] |
| Change transport mode | Suzhou | Not using car | WHO_5 Index | Not significant predictor of wellbeing in multivariate analysis | = | [14] |
|  |  |  |  |  |  |  |
| *Discourage car use* |  |  |  |  |  |  |
| 4% reduction of personal cars | Basel | Noise ↓ | Noise annoyance | .07↑ DALY /1000 | - | [6] |
| 4% reduction of personal cars | Basel | Noise ↓ | Sleep disturbance | .06↑ DALY /1000 | - | [6] |
| 4% reduction of personal cars | Basel | Noise annoyance↑ | Mental health item | probability of good wellbeing unchanged for city population  .6%↓ probability of good wellbeing for highly annoyed population | - | [16] |
| 10% reduction of personal cars | Rotterdam | Noise ↓ | Noise annoyance | ↓22 YLD | + | [12] |
| 10% reduction of personal cars | Rotterdam | Noise ↓ | Sleep disturbance | ↓36 (UI 20-51) YLD | + | [12] |
| Parking management | Kuopio | Parking at home | WHO_5 Index | Not significant predictor of wellbeing in multivariate analysis | = | [15] |
|  |  |  |  |  |  |  |
|  |  |  |  |  |  |  |
| *Multiple measures-transport* |  |  |  |  |  |  |
| Agreed traffic reduction measures  & strict diesel emission standards (vs 2010) | Basel | Noise annoyance↓ | Mental health item | .3%↑ probability of good wellbeing for city population  1.2%↑ probability of good wellbeing for highly annoyed population | + | [16] |
| Reduce traffic noise | Suzhou | Near busy road | WHO_5 Index | ↑ 2.9 (0.6 to 5.2)percentage points | - | [14] |
| Reduce traffic noise | Suzhou | Noise annoyance at home | WHO_5 Index | ↓ 5.3 (1.1 to 9.4)percentage points | + | [14] |

+ wellbeing improvement, + possible minor wellbeing improvement, = no change in wellbeing, - possible minor wellbeing deterioration, - wellbeing deterioration

Table A4c Effect of policies on economic wellbeing

| Policy | City | Reason for change in cost | Change | Sign | Ref |
| --- | --- | --- | --- | --- | --- |
| *ENERGY* |  |  |  |  |  |
| Domestic biomass burning | Stuttgart | Health effects of PM_2.5_ ↑ vs damage caused by higher CO_2_ emissions | Additional monetary impact of efficient boiler with dust filter €0.82m (0.29-2.28) | - | [1] |
| Domestic biomass burning | Thessaloniki | Chronic bronchitis, cardiovascular and respiratory diseases | €50m | -- | [1] |
| Reduce energy intense industry | Suzhou | Slow carbon emissions | Economic growth (US$ 15,840-22,260 per capita GDP) from 2005 to 2010 caused 47.3 million tons of carbon emissions | -- | [3] |
| *BUILDINGS* |  |  |  |  |  |
| Energy Efficiency Certification of buildings | Thessaloniki | Mortality & morbidity | >€1 bln | ++ | [1] |
| *TRANSPORT* |  |  |  |  |  |
| 50% electric vehicles | Thessaloniki | PM_10_↓ | €60.4m | ++ | [8] |
| 50% electric vehicles | Thessaloniki | PM_2.5_ ↓ | €49.1m | ++ | [8] |
| 50% electric vehicles | Thessaloniki | NO_2_↓ | €41.1m | ++ | [8] |
| 50% electric vehicles | Thessaloniki | C_6_H_6_↓ | €1.1m | ++ | [8] |
|  |  |  |  |  |  |
| New metro and increase in cars fuelled by diesel | Thessaloniki | PM_10_↓ | €56.6m | ++ | [8] |
| New metro and increase in cars fuelled by diesel | Thessaloniki | PM_2.5_ ↓ | €45m | ++ | [8] |
| New metro and increase in cars fuelled by diesel | Thessaloniki | NO_2_↓ | €37.7m | ++ | [8] |
| New metro and increase in cars fuelled by diesel | Thessaloniki | C_6_H_6_↓ | €1.0m | ++ | [8] |
| Measures to reduce noise | Kuopio | Health effects of noise | Noise contributes a third of the total burden of disease of the assessed sources in Kuopio. Cost impact of these health effects is around €18m/a. | ++ | [5] |
| *MULTIPLE -ANY* |  |  |  |  |  |
| Heat & power plant fuel peat & oil changed to wood,  biofuels for transport, building renovation | Kuopio | Health effects of PM_2.5_↓ | €1.5m (of €33m/a) | ++ | [5] |

Box A2 Noise exposure response functions

**Coronary Heart disease** *Basel [6]*

 8% increase in risk per increase of the weighted day-night noise level L _DN_ of 10 dB (A) within the range of approximately 52-77 dB (A) (5 dB-category midpoints). [17]

**Myocardial Infarction** for Lday: 57 – 80 dB(A) *Thessaloniki [8, 9]*

OR = 1.63 – 6.13*10^-4^*(Lday,16h)^2^ + 7.36*10^-6^*(Lday,16h)^3^

and

RR = (e ((OR-1)/10) * (Lday,16h) – 1) / e((OR-1)/10)*(Lday,16h) * BHE * EP

BHE: Background Health Effect i.e. 1.4/1000

EP: Exposed Population

**Wellbeing**

*Rotterdam [12] Basel [6]*

% Highly annoyed = 9.868*10^-4^ (Lden-42)3 - 1.436*10^-2^(Lden-42)^2^+ 0.5118 (Lden-42) [18]

% Highly sleep deprived = 20.8 – 1.05 * Lnight + 0.01486 *Lnight^2^ [19]

*Thessaloniki [8, 9]*

% of Population with Sleep Disturbance (SD)

%HSD = 20.8 – 1.05*Lnight + 0.01486*(Lnight)^2^ when Lnight: < 65 dB(A)

%SD = 13.8 – 0.85*Lnight + 0.01670*(Lnight)^2^ when Lnight: 45 – 65 dB(A)

%LSD = -8.4 + 0.16*Lnight + 0.01081*(Lnight)^2^ when Lnight: 0 – 45 dB(A)

Sleep annoyance due to road transport

 HA[%] = 0.5118*(Lden - 42) – 1.436*10^-2^*(Lden - 42)^2^ + 9.868*10^-4^*(Lden - 42)^3^

Box A3 Cross sectional wellbeing surveys in Suzhou and Kuopio

|  | Kuopio | Suzhou |
| --- | --- | --- |
| Date | December 2013 | August 2013 |
| Sampling strategy | A questionnaire was sent to a random sample of 2500 households in Kuopio. There were 680 responses. Additionally the survey was made available electronically and the link to the questionnaire was publicised via the internet, three organisational email listings, newspapers and radio. There were 102 additional responses from this, totalling 782 responses. | 775 face to face interviews were conducted with a random sample of residents. All residents approached took part. |
|  | | |
| Topics | | |
| Type | Details | |
| Wellbeing | WHO_5 wellbeing scale (feelings of cheerfulness, calm/relaxed, active/vigorous, fresh/rested, daily life filled with interest) and responses were converted to a score between 0 and 100 [20] | |
| Sociodemographics | Age, gender, SES | |
| Health | Physical and mental, satisfaction with health | |
| Environment | Satisfaction with work, housing, indoor home temperature, distance to green space, distance to a busy road, noise, household fuel, indoor and outdoor air quality and main mode of transport | |
| Relationships | Satisfaction with family life, social life and neighbours | |
| Psychosocial feelings of security | Comparisons of their life compared with other people, feeling safe and feeling in control- these issues may reflect how different environments become internalised | |

# References

1. Friedrich R, Kampffmeyer T, Torras Ortiz S: **Health impacts and greenhouse gas reductsion caused by using wood pellets for domestic heating in the city of Stuttgart** *URGENCHE WP6 Deliverable 6-3 Health and Wellbeing papers.* 2015.

2. Asikainen A, Savastola M, Parjala E, Kettunen T, Nittynen M, Tuomisto J: **URGENCHE WP10: health effect assessment of CO2 emission reduction methods in City of Kuopio**. City of Kuopio and THL; 2014.

3. Wang H, Wang Y, Wang H, Liu M, Zhang Y, Zhang R, Yang J, Bi J: **Mitigating greenhouse gas emissions from China's cities: Case study of Suzhou**. *Energy Policy* 2014, **68**(0):482-489.

4. Torras Ortiz S: **WP2 Energy**. *Urgenche Project Meeting.* Rotterdam; 2014.

5. Tuomisto J, Nittynen M, Parjala E, Asikainen A, Perez L, Trueb S, Jantunen M, Kunzli N, Sabel C: **Building-related health impacts in European and Chinese cities -scalable assessment method**. *URGENCHE WP4 Deliverable* 2015.

6. Perez L, Trueb S, Cowie H, Keuken M, Mudu P, Ragettli M, Sarigiannis DΑ, Tobollik M, Tuomisto J, Vienneau D *et al*: **Transport-related measures to mitigate climate change in Basel, Switzerland: a health effectiveness comparison study** *URGENCHE WP6 Deliverable 6-3 Health and Wellbeing papers.* 2015.

7. Keuken M, Jonkers S, Verhagen H, Perez l, Trueb S, Okkerse W-J, Liu J, Pan X, Zheng L, Wang H *et al*: **Impact on air quality of measure to reduce CO2 emissions from road traffic in Chinese and European cities**. *Atmospheric Environment* 2014, **98**:434–441.

8. URGENCHE Project Partners: **URGENCHE Project Final Report**. Edited by Sabel C, Bennett J; 2015.

9. Sarigiannis DΑ, Kontoroupis P, Chapizanis RN, Gotti A, Karakitsios SP: **Health impact assessment of the traffic related greenhouse gases (GHG) emission policies - the case study of Thessaloniki Greece**. *ICUH 11th International Conference on Urban Health.* Manchester, UK; 2014.

10. Sarigiannis DΑ, Karakitsios SP, Kermenidou M, Nikolaki S, Zikopoulos D, Semelidis S, Papagiannakis A, Tzimou R: **Total exposure to airborne particulate matter in cities: The effect of biomass combustion**. *Science of The Total Environment* 2014, **493**(0):795-805.

11. Sarigiannis DΑ, Kontoroupis P, Karakitsios SP, Nikolaki R, Chapizanis D: **Health impact assessment of the greenhouse gases (GHG) emission policies - the case study of Thessaloniki Greece**. *URGENCHE project meeting.* Rotterdam; 2014.

12. Tobollik M, Mudu P, Perez L, Trueb S, Cowie H, Keuken M, Tuomisto J, Vienneau D, Sabel C, Kunzli N: **Health impact assessment of transport policies in Rotterdam: decrease of total traffic and increase of electric car use**. *Environmental Research* In press.

13. Okkerse WJ, Willers S, van den Elshout S: **Health impact assessment of Rotterdam climate measures**. *URGENCHE project meeting.* Rotterdam; 2014.

14. Liu M, Huang Y, Hiscock R, Bi J, Sabel C: **Wellbeing impacts of climate change policies in Suzhou, China**. *URGENCHE WP6 Deliverable 6-3 Health and Wellbeing papers.* 2015.

15. Hiscock R: **What factors related to climate change mitigation and adaption policies are related to wellbeing in Kuopio Finland?** ; 2014.

16. Braubach B, Tobollik M, Mudu P, Hiscock R, Chapizanis D, Sarigiannis DΑ, Keuken M, Perez L, Martuzzi M: **Development of a quantitative methodology to assess the impacts of urban transport interventions and related noise on wellbeing**. *International journal of environmental research and public health* 2015, **12**(6):5792-5814.

17. Babisch W: **Updated exposure-response relationship between road traffic noise and coronary heart diseases: A meta-analysis**. *Noise and Health* 2014, **16**(68):1.

18. Miedema HM, C.G. O: **Annoyance from transportation noise: relationships with exposure metrics DNL and DENL and their confidence intervals**. *Environmental Health Perspectives* 2001, **109**(4):409-416.

19. Miedema HM, Passchier-Vermeer W, Vos H: **Elements for a position paper on night-time transportation noise and sleep disturbance**: TNO Inro; 2003.

20. **WHO-Five Well-being Index (WHO-5)** [<http://www.who-5.org/>]
