# Supplementary material for: Public health impacts of city policies to reduce climate change: findings from the URGENCHE EU-China project
Source: Environ Health. 2016 Mar 8;15(Suppl 1):25. doi: 10.1186/s12940-016-0097-0 (PMC4895602; doi:10.1186/s12940-016-0097-0)
Supplement: Additional file 2: — Peer review reports. (PDF 435 kb) [file 12940_2016_97_MOESM2_ESM.pdf]

Reviewer 1

Title: Public health impacts of city policies to reduce climate change: findings from the URGENCHE EU-China project

Authors: Sabel at al

Reviewer: Dr Zaid Chalabi, Social and Environmental Health Research Department, London School of Hygiene and Tropical Medicine, Keppel Street, London WC1H 9SH

This is a very interesting study and adds significantly to the literature in this area. The paper is very clearly written and well presented. It integrates the results of a major 7th European Framework Programme project on the public health impacts of city polices to reduce climate change (URGENCHE). It focusses on five European cities and two Chinese cities. The novel outputs of this study are the comparisons of the likely health co-benefits (or co-harms) in several cities of implementing realistic mitigation options in transport, housing energy efficiency and energy supply, and the inclusion of wellbeing in the co-benefits assessments.

For an otherwise a good paper, I have some comments and suggestions for the authors to consider.

1. The paper would be strengthened and be self-contained if the authors list the main assumptions underpinning the health impact calculations and therefore the findings. Although some of the findings of URGENCHE are in the public domain in peer-reviewed journals and readers would be able to refer to these publications, other findings are not in the public domain e.g. URGENCHE references [20], [23], [27], [31]-[33], [35] in the main text - and more importantly - most of the references cited in the results tables. I suggest that the authors include a table listing some of the key assumptions behind the health impact calculations of the analysed mitigation policies (see below for some details). This table could be included either in the main text or in the results tables.
2. For example, it was not clear whether the modelled mitigation policies were assumed to be implemented instantaneously (step change) in a city in 2020 (i.e. as a "thought experiment") or phased over time between 2010 and 2020.
3. What changes in indoor environmental exposures did the authors model in their assessment of housing energy efficiency policies? It seems they have considered $PM_{2.5}$ and PM_{10} only. I suggest that the authors point out that there are other health-relevant indoor environmental exposures which are also affected by housing energy efficiency measures but which they have not considered. These include radon, mould and indoor temperature (see e.g. Hamilton et al 2015).
4. The results tables give the health impacts in terms of a number of health metrics including DALYs. Using DALYs implies that the authors used the Comparative Risk Assessment (CRA) approach to calculate the health burdens. Did they use country-level 2010 WHO Global Burden of Disease (GBD) health and population profiles as the baseline for estimating the health impacts associated with changes in exposures? Am I correct that there is no information on city-specific GBD profiles which they could have used?
5. In relation to domestic heating policies Kuopio, the health measures that were used included "work loss days" and "Restricted activity days". It is unclear what is meant by these particularly as they seem to be unrelated to hospital admissions. Do they represent time off-work resulting from morbidity? If so, what where the cause-specific diseases? What type of exposure-response relationships were used to calculate the impacts?
6. It would be useful if the authors provide more information on how they calculated the impacts of changes in noise exposure. It seems that they considered its impact on wellbeing, mental health and physical health. Were these integrated into one impact figure? For example, in one of the cities (Basel), the health measure presented was "Mortality, annoyance, sleep disturbance".

What exposure-response relationships were used, particularly in relation to “annoyance” and “sleep disturbance” and did these form part of the DALY figure of 0.07 DALY per 1000? Furthermore is there a risk of double-counting of impacts? How would they differentiate the various measures which they used for noise exposure which seem to be inter-related: wellbeing, mental health, sleep disturbance and annoyance?

7. It is worth noting that there is a very recent epidemiological evidence on the association of road traffic noise in London with increased cardiovascular morbidity and mortality and all-cause mortality (Halonen et al, 2015).
8. Did the authors assume any time lags for health impacts resulting from changes in exposures associated with the mitigation policies? Some changes in health impact are likely to occur within a short period whilst others are likely to take much longer. For example, reduction in cardiac deaths associated with air pollution can take days to weeks whereas reduction in mental health (depression) associated with increased physical activity can take years (see e.g. Remais et al 2014). This would not be an issue if the time horizon of analysis is long but in this analysis it is about 10 years.
9. I would have liked to see some discussion on uncertainty. Naturally there are many sources of uncertainty in the health impact assessment of mitigation policies and it would be beyond this paper to consider and list all of them. There is for example uncertainty in the exposures, the exposure-response relationships, future population health profiles etc… Nevertheless it would be relevant to list some of the key uncertainties in the health impact calculations. These could include parametric and structural uncertainties in the models (e.g. see Remais et al 2014).
10. On a related matter, the results tables provide some quantitative figures of the impacts (the column marked “Change”) but there is no indication on the uncertainty in these impacts. It is not that clear how to interpret the “sign” column except to indicate the strength of the impact. Ideally 95% Confidence Intervals (or 95% Credible Intervals) should be calculated and presented, however I appreciate this would be a major undertaking and I do not propose that this is done (as matter of fact it was only done for the changes in the wellbeing index based on the analysis of the survey data). However some indication or discussion of the level of uncertainty (or confidence) in the calculated impacts would be useful.
11. Could the authors comment further on how policy makers would in practice use the outcomes of their study? Their study considered several criteria in evaluating the mitigation policies such as health, wellbeing, inequalities, CO_2 emissions, and economics. As the authors have found, and pointed out, the impacts on some criteria could be positive whilst the impacts on other criteria could be negative. It is not easy to see how policy makers can take into account conflicting impacts when evaluating mitigation policies without the use of some decision-support approach such as multi-criteria decision analysis (e.g. see Scrieciu et al (2014).
12. Finally I would like to draw the attention of the authors to the very recent Lancet Commission on health and climate change which also included collaboration from Europe and China (Watts et al 2015). It would be interesting if the authors compare their recommendations to those of the Commission in relation to mitigation policies for cities particularly on the mix of energy use and housing energy efficiency policies.

<u>References</u>

Halonen et al (2015) Road traffic noise is associated with increased cardiovascular morbidity and mortality and all-cause mortality in London. *European Heart Journal*. DOI: 10.1093/eurheartj/ehv216

Hamilton et al (2015) Health effects of home energy efficiency interventions in England: a modelling study. *BMJ Open.* DOI: 10.1136bmjopen-2014-007298.

Remais et al (2014) Estimating the health effects of greenhouse gas mitigation strategies: addressing parametric, model and valuation. *Environmental Health Perspectives* 122, 447-455.

Scrieciu et al (2014) Advancing methodological thinking in practice for development-compatible climate policy planning. Mitigation and Adaptation Strategies for Global Change 19, 261-288.

Watts et al (2015) Health and climate change: policy responses to protect public health. *Lancet* http://dx.doi.org10.1016/s0140-6736(15)60854-6

Reviewer 2

Review

Public health impacts of city policies to reduce climate change: findings from the URGENCHE EU-China project

A strength of this study is its naturalistic design. By that I mean the collaboration between researchers and city officials, to evaluate and report on policies that were chosen (by and large) by the cities themselves. This rather messy, particular and unpredictable process of policy development is what happens in the real world. However, the design is also a limitation, precisely because the policies that are reported here are particular to the cities in which they are implemented. This makes it difficult to move from the particular instance to what would apply elsewhere, and I feel the paper does not acknowledge sufficiently the generalizability problem. I provide particular instances below.

In many places the paper requires strong editing. Again, I provide some examples in my specific comments.

Abstract

"adopting policies to reduce private car use may have benefits for carbon dioxide reduction may have benefits for carbon dioxide and some health impacts". It would be helpful, given many readers will not proceed past the abstract, to explain what is meant by "some health impacts". Positive, negative? Large, small? What kinds of impacts?

"impact on ambient CO2 was limited" this is confusing – is this a reference to CO2 emissions? Or local air pollution (though CO2 is not really an issue in this sense)?

I feel the conclusion should tackle the generalizability issue (as above). The effectiveness and cost-effectiveness of policies depend very strongly on the context. For example, the impact of congestion charging in European cities with well-established infrastructure for walking, cycling and public transport will be quite different to the effects in cities in which people have few choices as to how they get around. You can't leave your car at home and catch the metro instead if there is no metro! Investments in greater energy efficiency in homes are likely to generate less value per dollar in densely settled cities with housing that is already high quality (and electricity is cheap), than in cities in which the housing stock is leaky and poor quality (and electricity is very expensive). So I suggest the findings (and the conclusions) need to be more directly tied to the particular circumstances and the particular cities in which the study was conducted.

"technological innovation" is an empty term – I suggest there is an explanation of what is meant here, and on what basis it is concluded that such innovation is the best bet for greenhouse gas reductions. (I'm not convinced that the body of the paper provides this evidence actually.)

what is “international cooperation for economic viability”?

Background

The last sentence in the first paragraph needs to be edited Should it be “an assessment”?

As I mentioned in my opening comments, it is a strength of the paper that it focuses on the policies that urban authorities can influence. However, there is no golden rule of governance. In different countries the distribution of powers varies widely. Perhaps this is true even within some countries. So I suggest that the authors add some text, to elaborate on what city councils and like bodies can and cannot “instigate in order to reduce GHG emissions”. This is not straightforward, because administration and funding are separated in many places. So in principle, city councils may be able to make decisions about road design and speed management, for instance, but the money to do this work comes largely from central government. Control of public utilities (power, water, waste) rests with cities in some instances, with regional authorities elsewhere, and with national government in other places. So, again, this is a report on a very particular set of cities, and whether and how far the findings relate to cities elsewhere is open to question.

“a society with high levels of well-being is likely to have low levels of inequalities”. Is this a normative statement, or does it refer to measurement and comparison? Some references are needed.

“China has the highest carbon dioxide emissions globally’. This is not true on a per capita basis, which (arguably) is a more relevant statistic in considerations of climate equity!

Methodology

The time frames applied in this study are very tight. One could argue that it is a tall order to seek demonstrable impacts by 2020. Infrastructure improvements have long lag times. Even large-scale social interventions may take effect over decades. The choice of end date reflects political timetables, I suspect. But the limitations, the fact that this study is essentially looking only at the potential for rapid returns, should be acknowledged.

Literature review

Why did the reviews examine “air pollution, noise **and/or** physical activity” It is the and/or bit that is difficult to understand.

Results

The reference to global emission reduction targets should specify the particular targets that the authors have in mind.

Would anyone expect city policies on transport and housing to reduce global emissions? Of course efficiency gains may be out-weighed by more houses/people/cars/consumption world-wide. This caveat applies to all policies and interventions, not just carbon reduction efforts made by urban authorities.

What is meant by "electricity needed for geothermal production reduces its efficiency"?

I suggest it would be helpful to include particular examples of transfers of emission sources from within the city boundaries to elsewhere.

Congestion charging – the effect of this policy is acutely sensitive to the starting point. I suggest there is a suitable qualifier included here (eg "in the cities that we studied, congestion charging ….").

How many power plants world wide rely on peat?

"our results may overestimate the effectiveness of biomass in emission reduction" – does this depend on the time scale of the carbon accounting?

"local policies were found to have limited effects on reducing local ambient air pollution". Were there differences between cities? My hunch is that effects may be different in Chinese cities where there is heavy use of coal and a rapidly growing vehicle fleet, and only limited public transport options, compared with many well-equipped European cities in which much of the electricity is generated by renewable sources.

"technology will reduce industrial emissions" – is this wishful thinking? What technologies do the authors have in mind? What certainty is there that potentially effective technologies will be applied as intended? Some commentators argue that the barriers to the green economy are political rather than technical, and that social change is the pre-requisite to emission reduction, rather than magic bullets fired by clever machines.

The use of biomass for home heating – surely the effects of such a switch depend on how it is done. The impacts on local air quality for example, depend on the kinds of burners that are installed, and their maintenance. There are high efficiency wood burners now available which release very little particulate pollution outdoors, and are essentially sealed from the indoor environment.

"measures to increase cycling (in Basel) increased trips by [only] 2%". This might be a large number of additional trips if the starting number was large! Also, it would be helpful to know what kind of measures were adopted.

Given the European readership, I suggest the paper includes a reference to increased indoor concentrations of radon as a possible, unwanted consequence of greater energy efficiency in homes. (Refer to the paper by Paul Wilkinson and others in the BMJ in 2014.)

Impact of GHG reduction policies on overall wellbeing

The marginal effect of home insulation on well-being is a very good example of the “it depends on the context” point I have made before. People living in leaky homes without adequate heating and without access to clean, cheap power will benefit much more from insulation than people living in better quality homes with good heating. But the tone of the discussion throughout suggests the findings from this particular sample of cities will apply everywhere.

“slowing the growth of polluting industries is likely to slow economic growth” This is contentious. Are we talking here about measures of economic growth that take account of the environmental and social costs of pollution? What about the economic opportunities of green growth? (See for instance the New Climate Economy Report from earlier this year.)

“high tech low emission industries with satisfying jobs” – why do these industries need to be “high tech”? Bicycle shops? Organic farming?

“positive economic impacts with the exception of slowing industrial emissions…” I challenge this conclusion, because I believe it is based on a flawed notion of economic progress (the externalities issue, as flagged above).

CO_2 emission reduction and possible co-benefits

“in-home biomass burning … would be significantly detrimental to health” – again, I think this conclusion depends on the technology used. I am not convinced that all biomass sources are health damaging.

“reducing car use would be likely to reduce CO_2 emissions and the impact on health, if in existence….” This is a clumsy way of putting it I suggest. Perhaps it would be more interesting if the authors described the circumstances in which reducing car use would not have an impact on health.

Policy implications

I have some suggestions which may strengthen these conclusions

“stringent national emission standards” is ambiguous. Does this refer to regulation of vehicle emissions, or to controls on greenhouse gas emissions? I suggest also that

“more effective” needs to be qualified. More effective in achieving what? CO_2 reductions? Or health improvements? Or both?

“International bodies need to encourage the development of new technologies” What technologies? I think this needs to be explained. Are the authors referring to cheap bicycles, carbon capture and storage, driverless cars, nuclear power plants ….?

“discouraging private car use … may have small health co-benefits” Again, I suggest this needs to be qualified. As I read the paper, the conclusion is that in some cities in this study sample, reductions in private car use had small health co-benefits. (But I know that elsewhere, well-reported in the literature, the health effects of shifting trips from private cars to other modes of transport are large.)

The final conclusion “academic teams can collaborate successfully with city partners” is a comment on the process that led to the publication, and is not supported by anything that has gone before. There is nothing in the paper that describes the process by which researchers and urban authorities worked together. There is no description of the problems faced, the differences between different cities and countries (which must have been substantial), or ways found to get around inevitable differences in time frames and world-views. There are no reflections on how the positive outcome that apparently was enjoyed here can be repeated elsewhere. This would be good material for another paper perhaps, but given that there is nothing to back up the claim of successful collaboration, I suggest it is dropped.

Alistair Woodward
